# Analysis of the spatio-temporal dynamics of a Rho-GEF-H1-myosin activator-inhibitor reaction-diffusion system

**DOI:** 10.1098/rsos.241077

**Published:** 2025-04-04

**Authors:** Kudzanayi Zebedia Mapfumo, Victor Ogesa Juma, Gulsemay Yigit, Gift Muchatibaya, Anotida Madzvamuse

**Affiliations:** ^1^ Mathematics Department, University of British Columbia, 1984 Mathematics Road, Vancouver, British Columbia V6T 1Z2, Canada; ^2^ Department of Mathematics, Faculty of Engineering and Natural Sciences, Bahçe¸ Sehir University, Istanbul, Turkey; ^3^ Department of Mathematics and Computational Sciences, University of Zimbabwe, Mt Pleasant, Harare, Zimbabwe; ^4^ Department of Mathematics and Applied Mathematics, University of Pretoria, Pretoria 0132, South Africa; ^5^ Applied Mathematics, University of Johannesburg, PO Box 524, Auckland Park 2006, South Africa

**Keywords:** reaction-diffusion, bifurcation analysis, Rho-GEF-Myosin signalling network, activator-inhibitor system, Turing diffusion-driven instability, travelling wave front

## Abstract

This study presents a detailed mathematical analysis of the spatio-temporal dynamics of the RhoA-GEF-H1-myosin signalling network, modelled as a coupled system of reaction-diffusion equations. By employing conservation laws and the quasi-steady state approximation, the dynamics is reduced to a tractable nonlinear system. First, we analyse the temporal system of ordinary differential equations (ODE) in the absence of spatial variation, characterizing stability, bifurcations and oscillatory behaviour through phase-plane analysis and bifurcation theory. As parameter values change, the temporal system transitions between stable dynamics; unstable steady states characterized by oscillatory dynamics; and co-existence between locally stable steady states, or co-existence between a locally stable steady state and a locally stable limit cycle. Second, we extend the analysis to the reaction-diffusion system by incorporating diffusion to the temporal ODE model, leading to a comprehensive study of Turing instabilities and spatial pattern formation. In particular, by adding appropriate diffusion to the temporal model: (i) the uniform steady state can be destabilized leading to the well-known Turing diffusion-driven instability (DDI); (ii) one of the uniform stable steady states in the bistable region can be driven unstable, while the other one remains stable, leading to the formation of travelling wave fronts; and (iii) a stable limit cycle can undergo DDI leading to the formation of spatial patterns. More importantly, the interplay between bistability and diffusion shows how travelling wavefronts can emerge, consistent with experimental observations of cellular contractility pulses. Theoretical results are supported by numerical simulations, providing key insights into the parameter spaces that govern pattern transitions and diffusion-driven instabilities.

## 1. Introduction

The movement of cells, in a process known as cell migration, is critical in various biological processes such as embryo development, bone formation, wound healing, immune defence and cancer progression [1–5]. This process involves a series of precisely orchestrated events that dictate the direction and efficiency of cell movement. The cell migration process begins with the establishment of directional polarity in a cell, characterized by a leading edge and a trailing end. This crucial step sets the stage for the subsequent dynamic events that propel the cell forward. Cyclic polymerization and depolymerization of actin filaments occur at the leading edge, accompanied by contraction of the trailing end, both of which are enriched with actomyosin [6–8]. The front of the cell protrudes and retracts in a cyclic manner until the protrusions known as lamellipodia are stabilized by attachment to the extracellular matrix [6,9]. Following this stabilization, the cell rear detaches, facilitating contraction and allowing the cell body to advance forward. Understanding these intricacies of cell migration is not only essential to unravel the mysteries of fundamental biological processes, but also holds significance in developing strategies to intervene in diseases, e.g. cancer, where aberrant cell migration contributes to metastasis [10].

Around 1995, researchers identified the Rho family of small GTPases as crucial contributors to the fundamental biochemical processes that govern cell migration [9,11,12]. Regardless of the specific type of cell migration, Rho GTPases constantly play a pivotal role, although their individual significance is influenced by factors such as the cellular environment, cell type and mode of migration [9]. Rho GTPases act as regulatory proteins, and their spatial distribution on the cell surface influences cell migration. Functioning as molecular switches, these proteins employ a simple biochemical mechanism to control complex cellular processes. They alternate between two conformal states: the active/ON state bound to guanosine triphosphate (GTP) and the inactive/OFF state bound to guanosine diphosphate (GDP) [11,13].

Rho GTPases, both in their active and inactive states, are present at the plasma membrane. However, inactive Rho GTPases are found in the cytosol bound to guanine dissociation inhibitors [13]. The cycling of Rho GTPases is meticulously regulated by guanine nucleotide exchange factors (GEFs) and GTPase activating proteins (GAPs). GEFs play a crucial role in promoting the transition to the active GTP-bound state by catalysing the exchange of GDP with GTP, while GAPs facilitate the reverse process, ensuring precise control over Rho GTPase activity [13]. In the active state (ON), Rho GTPases recognize target proteins and elicit a cellular response until GTP hydrolysis brings them back to the inactive state (OFF). To elucidate the specific contributions of certain interactions that influence cell migration patterns, scientists have put forth mathematical models, see [14–20]. In particular, recent studies have explored phase-field and free boundary models concerning cells assuming uniform adhesion at the front and contraction at the rear to understand the impact of actin polymerization and contractility induced by myosin on cell morphology [19,20].

**Figure 1.**
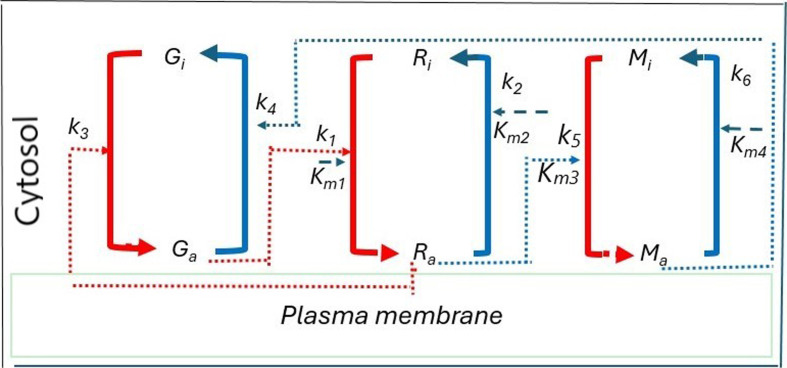
A schematic representation of the experimental model for the Rho-GEF-myosin signalling network from which mathematical equations are derived [21]. In this visual representation, the symbols $G_{a}$ , $R_{a}$ and $M_{a}$ denote the active states of GEF, Rho and myosin, while $G_{i}$ , $R_{i}$ and $M_{i}$ signify their respective inactive states. Reaction rates are represented as $k_{1}$ , $k_{2}$ , $k_{3}$ , $k_{4}$ , $k_{5}$ and $k_{6}$ , and the Michaelis–Menten constants are denoted as $K_{m_{1}}$ , $K_{m_{2}}$ , $K_{m_{3}}$ and $K_{m_{4}}$ . The broken red lines represent a positive feedback loop while blue lines represent a negative feedback loop.

This study seeks to investigate the spatio-temporal dynamics of a three-component RhoA (Rho)-GEF-H1 (GEF)-myosin signalling network. The study is conducted in two parts: first the temporal system of ordinary differential equations (ODE) is analysed and its temporal dynamics are fully characterized. The second part is to study the spatio-temporal dynamics of the full model whereby diffusion is added to the temporal model. The temporal system exhibits different dynamical regimes. As parameter values change, the system transitions through the experimentally observed dynamics [21], switching from stable steady states to unstable steady states characterized by oscillatory dynamics and then back to stable. In the presence of diffusion, the spatio-temporal model exhibit different dynamical behaviours. That is, when diffusion is added to the temporal model: (i) the uniform steady state can be destabilized leading to the Turing diffusion-driven instability (DDI); (ii) one of the uniform stable steady states in the bistable region can be driven unstable; (iii) while the other one remains stable, leading to the formation of travelling wave fronts; and (iv) a stable limit cycle can undergo DDI leading to the formation of spatial patterns. The spatio-temporal model that we propose for examination is based on the experimental findings documented in Kamps *et al.* [21] and Graessl *et al*. [22]. In this particular study, mathematical modelling proved to be an effective method for probing the causal connections between Rho, myosin and GEF, critical components involved in local cell contraction pulses [21,22]. To have a precise quantitative understanding of Rho activity dynamics linked to cellular contractility, the work by Kamps *et al*. [21] and Juma [23] developed a framework that outlines the essential biochemical reactions, as shown figure 1. During the analysis, the studies by Kamps *et al*. [21] and Graessl *et al*. [22] observed that GEF was recruited alongside Rho constructs and the Rho activity sensor, providing evidence of a causal connection between Rho activity and the localization of GEF to the cell membrane. Essentially, an increase in Rho activity at a specific location leads to enhanced GEF recruitment to the plasma membrane at the same site.

This system involves a positive feedback loop between Rho and GEF of the Lbc family and a negative feedback loop that inhibits GEF through myosin [21,22,24]. Active myosin prevents GEF from exchanging nucleotides, reducing Rho activation. Consequently, the activity states of Rho, GEF and myosin influence their association with the cell membrane [21]. To formulate the model based on these observations, the work by Kamps *et al*. [21], Graessl *et al.* [22] and Juma [23] took into account the following aspects and assumptions:

- GEF activation is facilitated by the conversion of inactive GEF ( $G_{i}$ ) to its active form ( $G_{a}$ ) through a non-enzymatic reaction with active Rho ( $R_{a}$ ) at the rate $k_{3}$ . This interaction is modelled using the mass action kinetics as shown in equation (1.2b);
- inhibition of GEF by myosin occurs through the direct binding of myosin and GEF to form a complex. This is achieved by the conversion of $G_{a}$ to $G_{i}$ facilitated by myosin at rate $k_{4}$ . The interaction is also represented using mass action kinetics, as shown in equation (1.2b);
- the conversion of Rho from an inactive state ( $R_{i}$ ) to an active state ( $R_{a}$ ) occurs through enzymatic processes at rate $k_{1}$ and is modelled using Michaelis–Menten kinetics with constant $K_{m_{1}}$ . Subsequently, $R_{a}$ is constitutively inhibited at rate $k_{2}$ using the Michaelis–Menten kinetics with a constant $K_{m_{2}}$ as shown in equation (1.2a); and
- the activation of myosin by $R_{a}$ involves the conversion of inactive myosin ( $M_{i}$ ) to active myosin ( $M_{a}$ ) at rate $k_{5}$ , captured using Michaelis–Menten kinetics with constant $K_{m_{3}}$ . The reaction is completed by constitutive inhibition of $M_{a}$ at rate $k_{6}$ , also modelled by the Michaelis–Menten kinetics with a constant $K_{m_{4}}$ as indicated in equation (1.2c).

To understand this system, Kamps *et al*. [21] and Juma *et al*. [23,25] developed various temporal models, based on different mathematical assumptions, in the form of ODEs to describe the dynamic changes in active GEF, Rho and myosin over time. From the reaction scheme shown in figure 1 the studies by Kamps *et al*. [21], Juma [23] and Juma *et al.* [25] formulated a model with variables $R_{a}(t)$ , $R_{i}(t)$ , $G_{a}(t)$ , $G_{i}(t)$ , $M_{a}(t)$ and $M_{i}(t)$ where t∈[0,T] and the subscript ${\;}_{i}$ denotes the inactive Rho, GEF and myosin species. The resulting model, without considering spatial effects, is given in terms of a system of nonlinear ODEs describing the rate of change of the concentrations of $R_{a}(t)$ , $R_{i}(t)$ , $G_{a}(t)$ , $G_{i}(t)$ , $M_{a}(t)$ and $M_{i}(t)$ , over time. The model is formulated by assuming that the total concentration of each species is constant (conserved) for all time. That is,


(1.1){Ra(t)+Ri(t)=RT=constant,Ga(t)+Gi(t)=GT=constant,Ma(t)+Mi(t)=MT=constant.

Following the mathematical translation of the biological assumptions described in (i) to (iv) above, the time evolution of $R_{a}(t)$ , $R_{i}(t)$ , $G_{a}(t)$ , $G_{i}(t)$ , $M_{a}(t)$ and $M_{i}(t)$ can be described by the following system of nonlinear ODEs:


(1.2a) $$\begin{array}{rl} & \left\{ \begin{array}{rl} \frac{dR_{a}}{dt}= & \frac{k_{1}G_{a}R_{i}}{K_{m_{1}}+R_{i}}-\frac{k_{2}R_{a}}{K_{m_{2}}+R_{a}},\\ \frac{dR_{i}}{dt}= & \frac{-k_{1}G_{a}R_{i}}{K_{m_{1}}+R_{i}}+\frac{k_{2}R_{a}}{K_{m_{1}}+R_{a}}, \end{array}\right. \end{array}$$

(1.2b) $$\begin{array}{rl} & \left\{ \begin{array}{rl} \frac{dG_{a}}{dt}= & k_{3}R_{a}G_{i}-k_{4}M_{a}G_{a},\\ \frac{dG_{i}}{dt}= & -k_{3}R_{a}G_{i}+k_{4}M_{a}G_{a}, \end{array}\right. \end{array}$$

(1.2c) $$\begin{array}{rl} & \left\{ \begin{array}{rl} \frac{dM_{a}}{dt}= & \frac{k_{5}R_{a}M_{i}}{K_{m_{3}}+M_{i}}-\frac{k_{6}M_{a}}{K_{m_{4}}+M_{a}},\\ \frac{dM_{i}}{dt}= & -\frac{k_{5}R_{a}M_{i}}{K_{m_{3}}+Mi}+\frac{k_{6}M_{a}}{K_{m_{4}}+M_{a}}. \end{array}\right. \end{array}$$

To close the system, non-negative initial conditions: $R_{a}(0)=R_{0};$ $G_{a}(0)=G_{0};$ $M_{a}(0)=M_{0};$ $R_{i}(0)=R_{i0};$ $G_{i}(0)=G_{i0};$ and $M_{i}(0)=M_{i0}$ , are prescribed. Applying mass conservation in equation (1.1), the ODE system (1.2a) reduces to a system of three nonlinear ODEs given by:


(1.3a) $$\begin{array}{rl} \frac{dR_{a}}{dt}= & \frac{k_{1}G_{a}(R_{T}-R_{a})}{K_{m1}+(R_{T}-R_{a})}-\frac{k_{2}R_{a}}{K_{m2}+R_{a}}, \end{array}$$

(1.3b) $$\begin{array}{rl} \frac{dG_{a}}{dt}= & k_{3}R_{a}(G_{T}-G_{a})-k_{4}G_{a}M_{a}, \end{array}$$

(1.3c) $$\begin{array}{rl} \frac{dM_{a}}{dt}= & \frac{k_{5}R_{a}(M_{T}-M_{a})}{K_{m3}+(M_{T}-M_{a})}-\frac{k_{6}M_{a}}{K_{m4}+M_{a}}. \end{array}$$

The findings of the ODE model derived, indicate a correlation between the transition from stable to oscillatory dynamics and vice versa, with lower concentrations of total GEF leading to oscillatory dynamics and at higher concentrations of GEF resulting in a return to stable dynamics. To address the irregular oscillations observed in experiments, Kamps *et al*. [21] devised a computational framework based on a system of stochastic differential equations.

To fully analyse the spatio-temporal activity dynamics, it is essential to establish a connection between reaction kinetics and transport processes such as diffusion, as emphasized by Gierer & Menhardt [26,27]. Consequently, Kamps *et al*. [21] extended the stochastic differential system to include spatial variations and conducted numerical simulations. The spatio-temporal model of six species was solved numerically using cellular automata. Although cellular automaton excels in tackling specific problem types, particularly those featuring discrete and localized interactions, they may not be the optimal choice for modelling continuous and complex systems that require a more precise representation of spatial patterns, as noted by Evans [28] and Wolfram [29]. In this context, we conducted a spatio-temporal analysis of the model using partial differential equations (PDEs) of reaction-diffusion type to explore its spatio-temporal dynamics. In this. article, our goal is to explore, analytically and computationally, the effects of model reduction using mass conservation as well as quasi-steady state approximation on the spatio-temporal dynamics of the Rho-GEF-H1-myosin signalling network.

Hence, the structure of this article is as follows. In §2, taking advantage of current insights from the model proposed in Kamps *et al*. [21] and Juma *et al*. [25], we apply experimentally derived conclusions to justify the formulation of a coupled nonlinear system of ODEs. Within the same section, we introduce diffusion and non-dimensionalize the reaction-diffusion system, paving the way for carrying out mathematical analysis of the model system. The analysis is divided into two parts: first we establish the positivity of solutions for the ODE system and then we study the long-term behaviour of the ODE system around uniform critical points. This is done by employing a numerical bifurcation analysis. Theoretical results are supported by numerical simulations. The second part involves the addition of diffusion to the temporal ODE system, to study under what conditions on the model parameters does the model exhibit Turing diffusion-driven instability. In the case where non-Turing dynamics emerge, we use numerical simulations to probe the type of solutions the reaction-diffusion might be able to exhibit. Finally, in §3, we discuss the results obtained and draw conclusions.

## 2. Material and methods

### 2.1. Quasi-steady state approximation

The quasi-steady state approximation is a method used in chemical kinetics to simplify the analysis of reaction mechanisms, particularly in multistep reactions where specific intermediates in the reaction pathway are produced and consumed much more rapidly than others [30–32]. The concept underlying the QSSA is that certain intermediate reactions can be considered to be in a ‘quasi-steady state’ owing to their formation and consumption rates being significantly faster than the rates of other reactions in the system [33,34].

Experimental results show that myosin activation occurs approximately 20−40 s after the onset of Rho and GEF activities [21,22]. This indicates that Rho and GEF activities occur on a shorter timescale compared with myosin activation. Therefore, we assume that GEF reaches a quasi-steady state relative to the slower dynamics of myosin activation. By doing so, we calculate the steady-state for GEF in equation (1.3*a*), setting the left-hand side of the GEF equation to zero. Substituting this into equation (1.3*a*), incorporating diffusion, we non-dimensionalise the system. Table 1 shows the physical model parameters and their dimensional quantities. See Appendix A for the non-dimensionalisation process. Hence, non-dimensionalisation results in the following system of reaction-diffusion equations (RDEs):

**Table 1.** Model parameters together with their corresponding physical units. (U represents concentration measured in molecules/cell while s represents time in seconds).

parameter	description	units	base value	reference
$G_{T}$	*G* total concentration	U	varies	[21,25]
$k_{1}$	*R* activation rate	${\text{s}}^{-1}$	0.0966−13.049	[21,25]
$k_{2}$	*R* inhibition rate	$\text{U}{\text{s}}^{-1}$	0.0652−2.160	[25]
$k_{3}$	*G* activation rate	${\text{U}}^{-1}{\text{s}}^{-1}$	0.723−3.34	[21,25]
$k_{4}$	*G* inhibition rate	${\text{U}}^{-1}{\text{s}}^{-1}$	1−14.9	[21,25]
$k_{5}$	*M* activation rate	${\text{s}}^{-1}$	0.0472−1	[25]
$k_{6}$	*M* inhibition rate	$\text{U}{\text{s}}^{-1}$	0.00933−0.1	[25]
$K_{m1}$	Michaelis–Menten constant for *R* activation	${\text{U}}^{-1}$	0.0886−2.43	[21,25]
$K_{m2}$	Michealis–Menten constant for *R* inhibition	U	0.0741−0.564	[21,25]
$K_{m3}$	Michaelis–Menten constant for *M* activation	U	0.0033−0.5	[25]
$K_{m4}$	Michaelis–Menten constant for *M* inhibition	U	0.258−1.83	[21,25]
$R_{T}$	*R* total concentration	U	0.443−1	[25]
$M_{T}$	*M* total concentration	U	1−1.24	[25]


(2.1) $$\begin{array}{r} \left\{ \begin{array}{l} \left\{ \begin{array}{l} \frac{\partial u}{\partial t}=\gamma f(u,v)+\nabla^{2}u,\\ \;\;\;\;\;\;\;\;\;\;\;\;\;\;\;\;\;\;\;\;\;\;\;\;\;\;\;\;\;\;\;\;\;\;\;\;\;\;\;\;\;\;\;x\in \Omega ,\;t>0,\\ \frac{\partial v}{\partial t}=\gamma g(u,v)+d\nabla^{2}v, \end{array}\right.\\ -(n\cdot \nabla u)=0,\;-d(n\cdot \nabla v)=0,\;\;x\in \partial \Omega ,\;t>0,\\ u(x,0)=u_{0}(x),\;v(x,0)=v_{0}(x),\;\;\;\;\;x\in \Omega ,\;t=0, \end{array}\right. \end{array}$$

where


(2.2) $$\begin{array}{lcr} & \left\{ \begin{array}{c} f(u,v)=\frac{a_{1}u(a_{2}-u)}{\left(a_{3}+a_{2}-u)(a_{4}v+u\right)}-\frac{u}{a_{5}+u},\\ g(u,v)=\frac{u(a_{6}-v)}{a_{7}+a_{6}-v}-\frac{v}{a_{8}+v}. \end{array}\right. & \end{array}$$

Throughout our analysis, we will consider the dimensionless model (2.1) together with its reaction kinetics (2.2). We will monitor and vary three parameters: $a_{1}$ , $a_{2}$ and $a_{6}$ since they exhibit a direct proportionality to their experimentally measured counterparts: $G_{T}$ , $R_{T}$ and $M_{T}$ , respectively, representing the total concentration of Rho, GEF and myosin. To elaborate, we consider $a_{1}$ as the bifurcation parameter, analogous to the role played by $G_{T}$ in the dimensional model as well as in experiments. As for $a_{2}$ and $a_{6}$ , they serve as the upper bounds for the non-dimensional system, mirroring the representation of $R_{T}$ and $M_{T}$ in the dimensional model. Experimentally, it was observed that the system transitions through stable, oscillatory and stable dynamics when the total GEF-H1 concentration ( $G_{T}$ ) is varied. Hence, it is natural to take $G_{T}$ as a bifurcation parameter. Having described the mathematical model of Rho-GEF-myosin dynamics and non-dimensionalized it, we proceed to explore the spatio-temporal dynamics of system (2.1), by first considering the analysis in the absence of diffusion and then in the presence of diffusion.

### 2.2. Mathematical analysis of the temporal system

Next, we analyse the temporal dynamics of the ODE part of system (2.1) for positive invariance, evaluate the stability of the uniform steady state, and investigate the characteristics of the phase planes. In the absence of diffusion, system (2.1) satisfies


(2.3) $$\begin{array}{lcr} & \left\{ \begin{array}{c} \frac{du}{dt}=\gamma f(u,v),\;\text{and}\;\frac{dv}{dt}=\gamma g(u,v), \end{array}\right. & \end{array}$$

with positive initial conditions $u(0)=u_{0}$ and $v(0)=v_{0}$ , and f(u,v) and g(u,v) as defined in equation (2.2). For the positive invariance and linear stability analysis in the absence of diffusion, see appendix B. Here, since we want to study the effect of adding diffusion to a uniform steady state in different dynamic regions, we start by summarizing the stability and bifurcation analysis results of the ODE system. Since there exists a diverse set of parameters with distinct dynamics, we present the results considering (i) the transition from stable to oscillatory, and (ii) the transition between stable, bistable and oscillatory dynamics.

#### 2.2.1. Numerical bifurcation, phase plane analyses and numerical simulation results in the stable and oscillatory region

**Figure 2.**
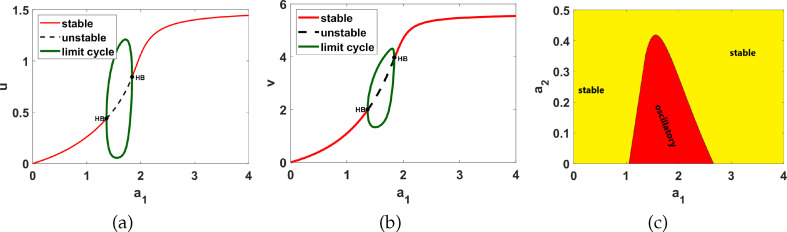
Numerical bifurcation results corresponding to system (2.3) with set 1 parameter values as listed in table 6. Plots (a) and (b) represent one-parameter bifurcation diagrams with bifurcation parameter $a_{1}$ . HB stands for the Hopf bifurcation point, and the dashed black line represents the values of u and v in the unstable region, while the solid red line represents the values of u and v in the stable region. Hopf bifurcation points occur at $a_{1}=1.365$ and $a_{1}=1.839$ . The green loop indicates the upper and lower limits of the resultant limit cycle. (c) represents the two-parameter bifurcation diagram with bifurcation parameters $a_{1}$ and $a_{5}$ . The red region represents the oscillatory region, while the lime region represents the stable region.

We conduct numerical bifurcation analysis of system (2.3) using XPPAUTO, a freely downloadable software program accessible at XPPAUTO [35] with $a_{1}$ as the bifurcation parameter and set 1 parameter values as listed in table 6. As shown in figure 2, the model exhibits up to two dynamical regimes when the parameter $a_{1}$ is varied. These results mirror those observed in experiments [21] and other published work [23,25]. Additionally, a two-parameter bifurcation diagram is generated by plotting $a_{1}$ against $a_{5}$ , as illustrated in figure 2c. In this diagram, the oscillatory region is highlighted in red. We select $a_{1}$ values within each of the three dynamical regimes and plot phase planes and corresponding phase portraits to illustrate the observed dynamics. We numerically calculated uniform steady states for each $a_{1}$ value together with set 1 parameter values from table 6. Uniform steady states corresponding to each region are computed and labelled $P_{11}$ , $P_{12}$ and $P_{13}$ in the phase planes and portraits shown in table 2, their stability analysed to validate the bifurcation analysis results.

**Table 2.** An illustration of nullcline configuration, phase portraits around the steady states ***P***_**11**_, ***P***_**1**_ and ***P***_**13**_, and the analysis for the possibility of DDI for system (2.3).

phase plane/nullcline configuration	phase portrait near the steady state	nature of steady states	possibility of DDI
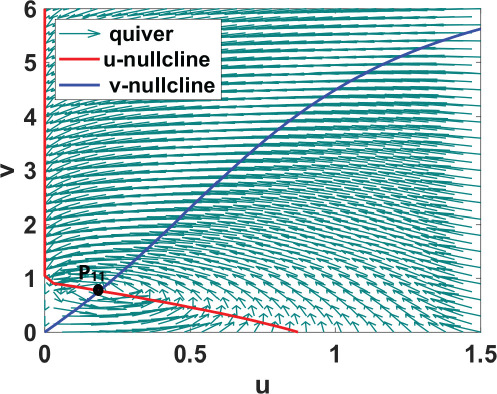	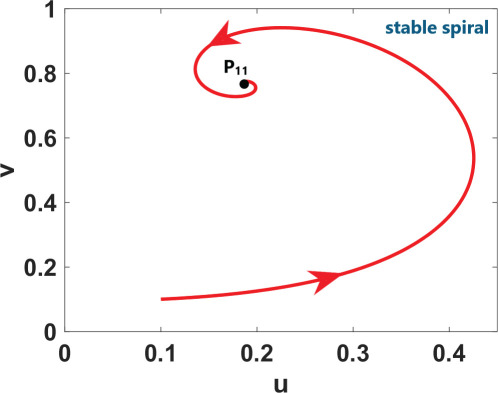	**steady state** ***P***_11_(018674, 0.7666) **eigenvalues** at ***P***_11_−0,184−0,395*i*−0,184+0,395*i*	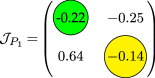 DDI not possible since *P*_11_ is stable and $f_{u}<0$ and $g_{v}<0$ . Refer to appendix B.
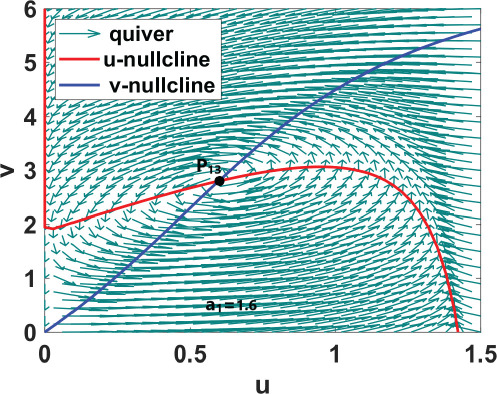	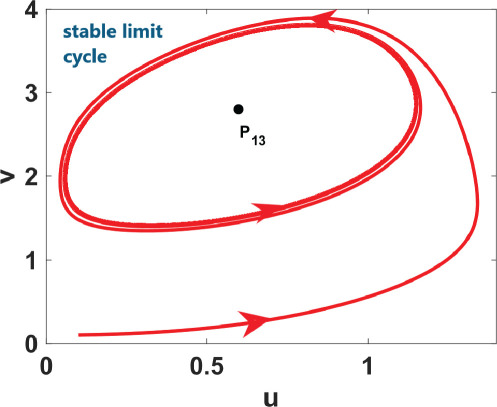	**steady state** ***P***_13_(0.59759, 2.8004) **eigenvalues** at (***P***_13_) 0.025−0.231*i* 0.025+0.231*i*	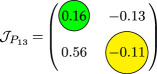 DDI not possible since *P*_13_ is unstable, that is, $f_{u}+g_{v}>0$ and $f_{u}g_{v}-f_{v}g_{u}>0$ . Refer to appendix B.
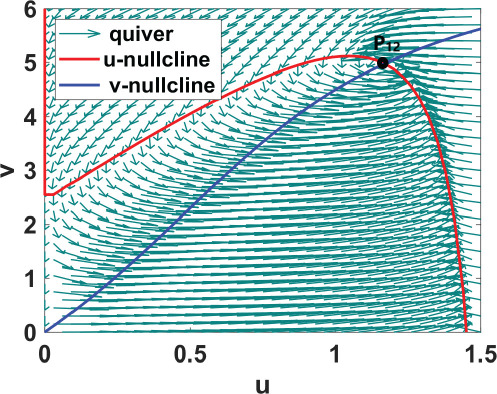	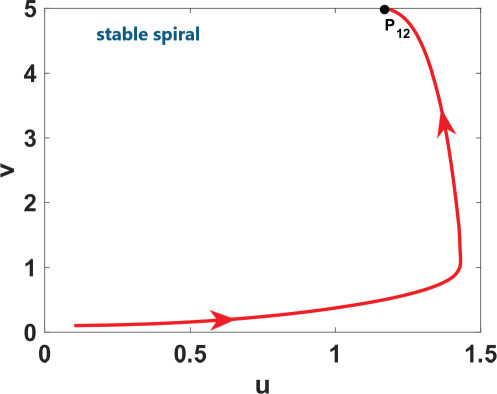	**steady state** ***P***_12_(1.168, 4.9796) **eigenvalues** at (***P***_12_) −0.193− 0.182*i*−0,193+0.182*i*	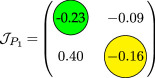 DDI not possible since *P*_12_ is stable and $f_{u}<0$ and $g_{v}<0$ . Refer to appendix B.

**Figure 3.**
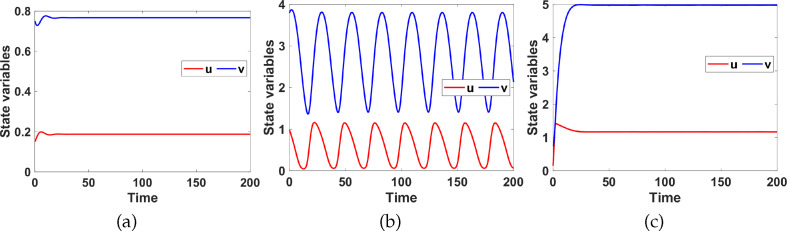
Numerical simulations showing temporal evolution profiles of u(t) and v(t) corresponding to system (2.3), with set 1 parameter values as listed in table 6 with (a) $a_{1}=0.5$ . (b) $a_{1}=1.5$ . (c) $a_{1}=2$ .

Table 2 shows a summary of numerical analysis results in the case of transition between stable and oscillatory dynamics. To validate the results of the numerical bifurcation analysis, we simulate system (2.3) for the values of $a_{1}$ that correspond to the stable, oscillatory and stable regions. The results are shown in figure 3. For small values of $a_{1}$ , the system has a unique uniform steady state which is globally asymptotically stable (G.A.S) (see figure 3a). As the value of $a_{1}$ increases, the uniform steady state becomes unstable, and the system exhibits periodic solutions arising from a Hopf bifurcation (see figure 3b). Increasing $a_{1}$ further returns the system to stable dynamics (see figure 3c).

#### 2.2.2. Phase plane, bifurcation analyses and numerical simulation results in the stable, oscillatory and bistable regions

Previously, we have shown that simulating system (2.3) using set 1 parameter values from table 6 generates three dynamical regimes for different $a_{1}$ values. These dynamics replicate what was observed in experiments [21]. Here, we show that by simulating system (2.3) using set 2 parameter values in table 6, up to five dynamical regions are generated. To analyse these dynamical behaviours, we perform numerical bifurcation, phase plane analysis and numerical simulations. Initially, we perform a two-parameter numerical bifurcation analysis with $a_{1}$ and $a_{5}$ as bifurcation parameters. Our decision to focus on the $a_{1}$ – $a_{5}$ parameter combination is informed by the experimentally observed activation of Rho which corresponds to the non-dimensional u variable. Specifically, $a_{1}$ describes positive feedback activation of u , while an increase in $a_{5}$ leads to a lower constitutive inhibition and a higher negative feedback of u . The results of the bifurcation analysis divide the parameter space into five dynamical regions marked I–V, as illustrated in table 3. In all of our analysis and simulations, we select fixed values of $a_{5}$ and vary $a_{1}$ appropriately. We perform a one-parameter bifurcation analysis with $a_{1}$ serving as the bifurcation parameter for $a_{5}=0.5$ , $a_{5}=1.5$ and $a_{5}=2$ . The results of the one-parameter bifurcation analysis are shown in figure 5.

**Table 3.** Pairwise parameter spaces and their corresponding illustration in figure 4.

space	$\left(a_{3},a_{5}\right)$	$\left(a_{3},a_{7}\right)$	$\left(a_{5},a_{8}\right)$	$\left(a_{1},a_{5}\right)$	$\left(a_{5},a_{7}\right)$	$\left(a_{4},a_{5}\right)$	$\left(a_{1},a_{7}\right)$	$\left(a_{4},a_{8}\right)$	$\left(a_{3},a_{8}\right)$
figure 4	(a)	(b)	(c)	(d)	(e)	(f)	(g)	(h) and (i)	

**Figure 4.**
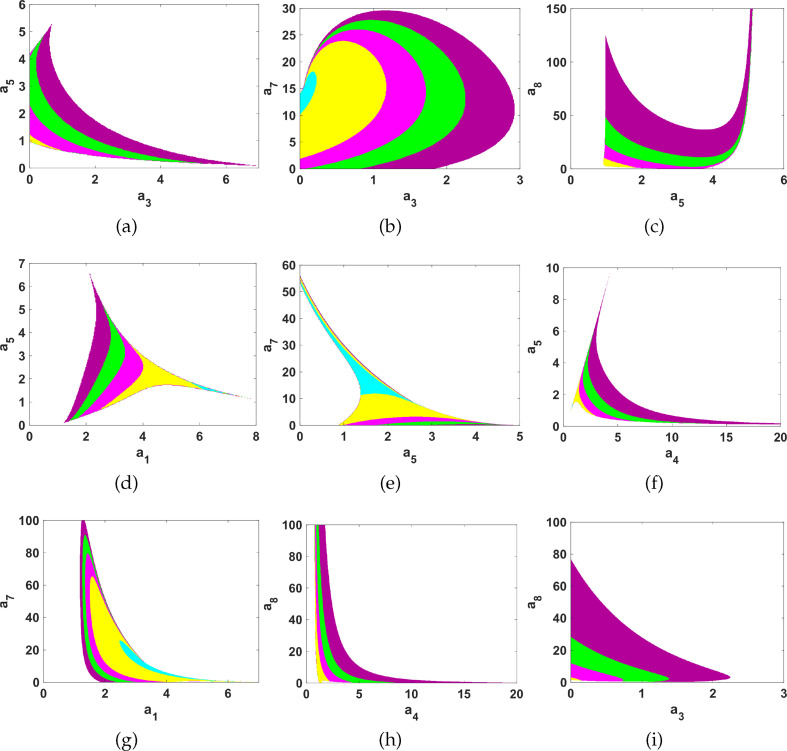
Turing instability regions in selected pairwise parameter planes for different d values. All other parameters remain fixed, as indicated in table 6. The explanation of the regions is shown in table 4.

**Table 4.** Key to parameter spaces in figure 4 for selected d values.

selected values of the diffusion coefficient	colour coding of the parameter spaces shown in figure 4
d=10	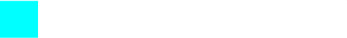
d=50	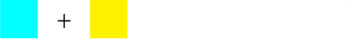
d=100	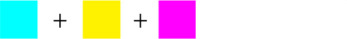
d=200	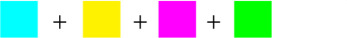
d=500	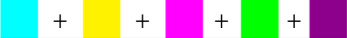

**Figure 5.**
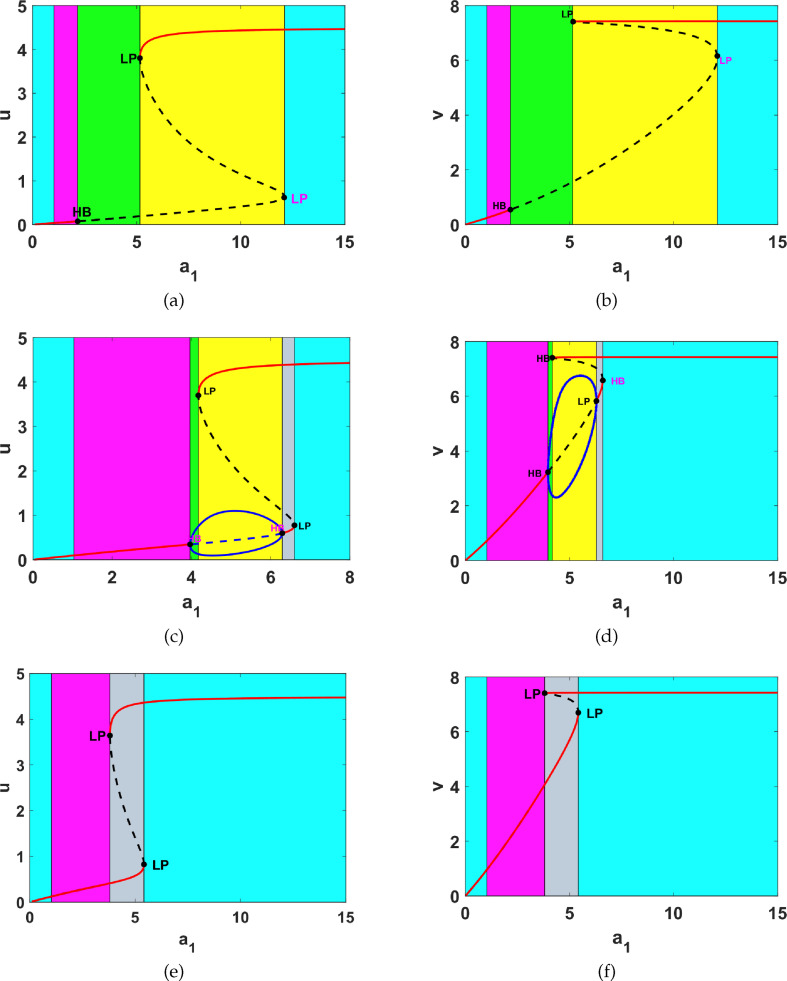
One parameter bifurcation diagrams corresponding to (a) $u^{*}$ against $a_{1}$ for $a_{5}=0.5$ , (b) $v^{*}$ against $a_{1}$ for $a_{5}=0.5$ , (c) $u^{*}$ against v for $a_{5}=1,5$ , (d) $v^{*}$ against $a_{1}$ for $a_{5}=1.5$ and (e) $u^{*}$ against $a_{1}$ for $a_{1}=2$ (f) $v^{*}$ against $a_{1}$ for $a_{5}=2$ . In all diagrams, LP and HB represent fold and Hopf bifurcation points, respectively.

**Figure 6.**
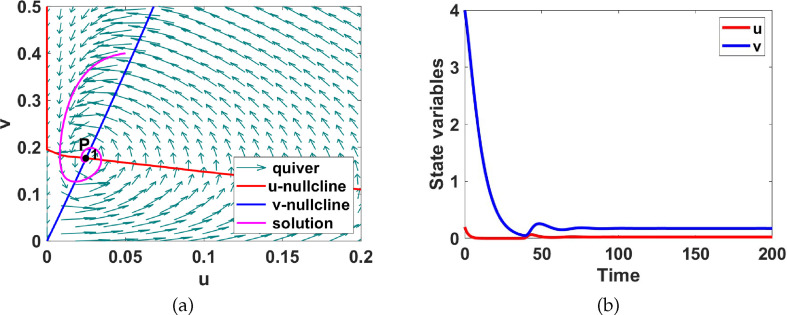
(a) Phase plane diagram showing that the uniform steady state **$P_{1}$** is globally asymptotically stable and, (b) the u(t) and v(t) time evolution for system (2.3) corresponding to region I of figure 11 . Here $a_{1}=0.2$ and $a_{5}=2$ .

**Figure 7.**
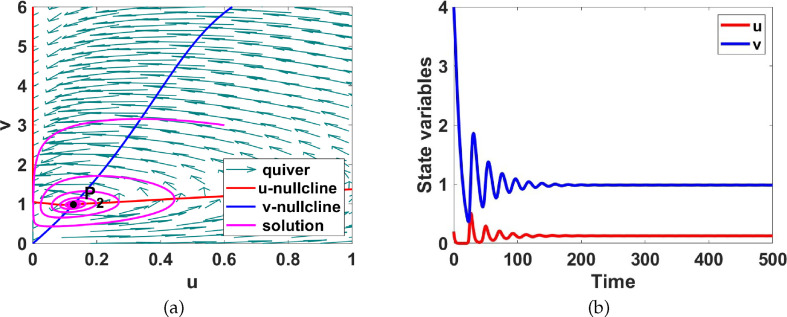
(a) Phase plane diagram showing that the uniform steady state $P_{2}$ is globally asymptotically stable and, (b) the u(t) and v(t) time evolution for system (2.3) corresponding to region II shown in figure 11, Here $a_{1}=2$ and $a_{5}=1$ .

**Figure 8.**
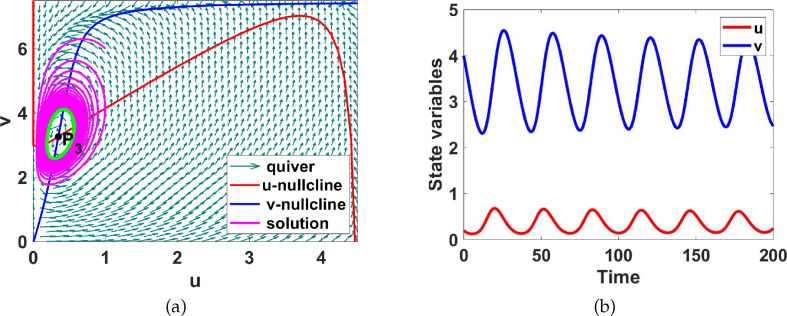
(a) Phase plane diagram showing the limit cycle around the uniform steady state $P_{3}$ , coloured green. (b) u(t) and v(t) time evolution for system (2.3) corresponding to region III of figure 11. Here $a_{1}=4$ and $a_{5}=1.5$ .

**Figure 9.**
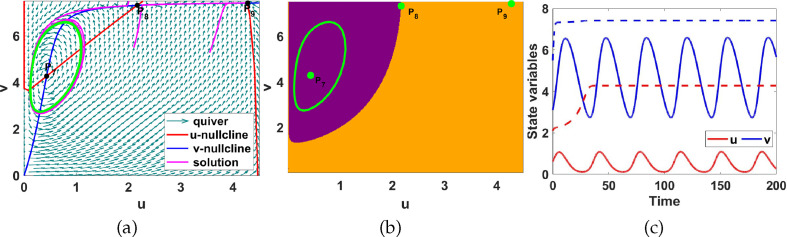
(a) Phase plane diagram showing coexistence of a L.A.S limit cycle (green loop) and L.A.S uniform steady state, $P_{9}$ , (b) the basin of attraction for the limit cycle around the uniform steady state $P_{7}$ and the steady state $P_{9}$ , and (c) the u(t) and v(t) time evolution for system (2.3) corresponding to region V of figure 11. Here $a_{1}=5$ and $a_{5}=1.5$ together with initial conditions (0.61,3.1) and (2.1,5.5) .

As seen in figure 5, for some parameter values, system (2.3) admits up to five different dynamical behaviours: in figure 5a, with $a_{5}=0.5$ fixed; for small values of $a_{1}$ , the uniform steady state is unique and G.A.S, which cannot be destabilized in the presence of diffusion (see region I and figure 6). As $a_{1}$ increases, the uniform steady state also slowly increases, remaining stable; however, this type of uniform steady state can be destabilized by diffusion, and for appropriate diffusion parameter values, the reaction-diffusion is capable of generating Turing patterns (see region II and figure 7). Furthermore, increasing $a_{1}$ makes the uniform steady state unstable, leading to periodic solutions arising from the Hopf bifurcation (see region III and figure 8). The system undergoes a fold bifurcation with increasing $a_{1}$ leading to the co-existence of three uniform steady states (one stable, a saddle and an unstable spiral), and therefore the uniform steady state is locally asymptotically stable (L.A.S) and co-exists with an L.A.S. limit cycle that surrounds a spiral. The saddle acts as a switch that determines which dynamics are observed (see region V and figure 9). Further increase in $a_{1}$ returns the system to stability.

**Figure 10.**
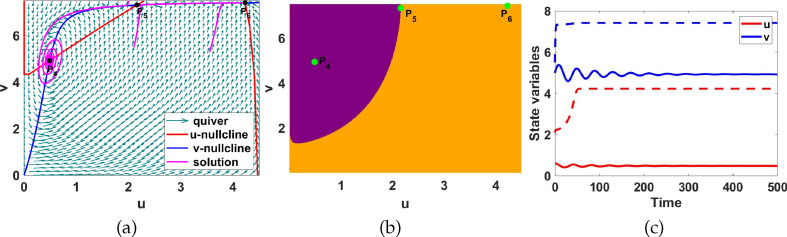
(a) Phase plane diagram showing coexistence of three uniform steady states, $P_{4}$ , $P_{5}$ and $P_{6}$ , (b) the basin of attraction for the uniform steady states $P_{4}$ and $P_{6}$ , and (c) the u(t) and v(t) time evolution for system (2.3) corresponding to region IV, shown in figure 11. Here $a_{1}=4.3$ and $a_{5}=2.1$ . The initial conditions for bistability are (0.6, 5) and (2.1, 5.5).

For $a_{5}=1.5$ , system (2.3) admits all dynamics (I to V), with the added dynamics from the case of $a_{5}=0.5$ being the existence of bistable dynamics (see region IV and figure 10). In this case, there is the co-existence of two stable uniform steady states, separated by a saddle, which acts as a switch, determining to which stable state the system converges.

For $a_{5}=2$ , system (2.3) loses two dynamics associated with regions III and IV as $a_{1}$ varies. Therefore, as $a_{1}$ varies, the system moves from having a stable uniform steady state for small values of $a_{1}$ to co-existence of three uniform steady states; two stable uniform steady states separated by a saddle point, and then back to stable dynamics at higher values of $a_{1}$ .

#### 2.2.3. Numerical simulations of the ODE temporal model across the different dynamic regions

Here, we perform numerical simulations to illustrate the dynamical regions identified in figure 5. By selecting the parameters $a_{1}$ and $a_{5}$ from each region, we plot the phase plane diagrams and the numerical simulation results of system (2.3) to validate the results of the bifurcation analysis.

**Figure 11.**
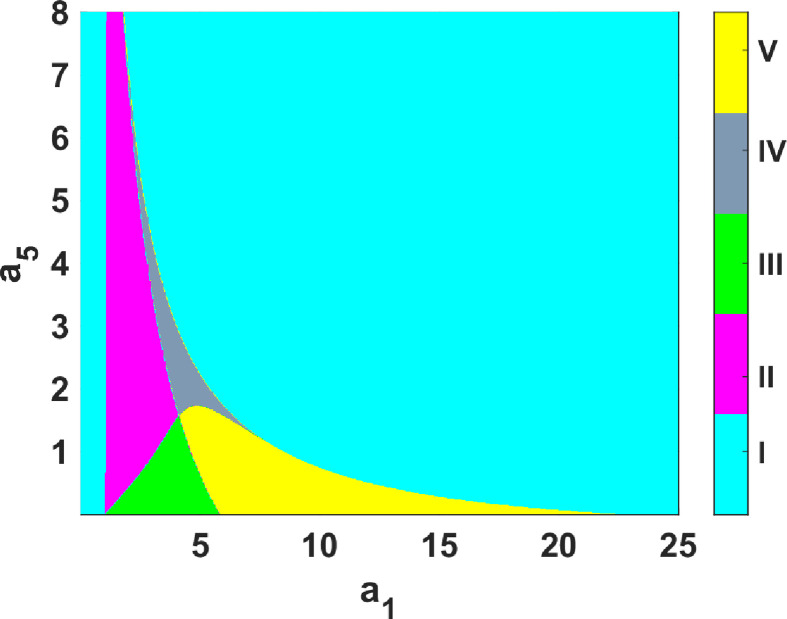
Two-parameter numerical bifurcation analysis results corresponding to system (2.3) with $a_{1}$ and $a_{5}$ as bifurcation parameters and set 2 parameter values as listed in table 6. I, single non-Turing uniform steady state; II, single Turing-type uniform steady state; III, single unstable uniform steady state; IV, three uniform steady states with one stable and the other unstable (bistable region); V, three uniform steady states with two unstable and one stable.

**Region I.** This region coloured cyan is characterized by a unique stable uniform steady state $P_{1}$ with spiral character. The Jacobian matrix for the system evaluated at the uniform steady state has the sign pattern $\left[\begin{array}{cc} - & +\\ + & - \end{array}\right]$ and hence does not admit Turing instability. For illustrative purposes, we pick parameters $a_{1}=0.2$ , and $a_{5}=2$ , with the other parameters fixed as listed in set 2 of table 6 and numerically compute the uniform steady state $P_{1}=(0.024877,0.17634)$ . We then generate the phase plane diagram shown in figure 6a as well as the solution for t∈[0,200] to validate the results of the bifurcation analysis obtained in figure 11.

**Region II.** This region in magenta is characterized by a unique stable uniform steady state $P_{2}$ with a spiral character. The Jacobian matrix evaluated at the uniform steady state has the sign pattern $\left[\begin{array}{cc} + & -\\ + & - \end{array}\right]$ . The system with added diffusion is capable of generating Turing patterns [36]. To illustrate the temporal dynamics, we select $a_{1}=2$ and $a_{5}=1$ from the region and compute the uniform steady state $P_{2}$ . The phase plane diagram and the time evolution of the solution to validate the results of the bifurcation analysis obtained in figure 11 are shown in figure 7. More details on the Turing analysis are discussed in appendix C.

**Region III.** The area highlighted in green colour contains a unique unstable uniform steady state $P_{3}$ , where the Jacobian matrix exhibits a specific sign pattern of $\left[\begin{array}{cc} + & -\\ + & - \end{array}\right]$ . The system (2.3) is conserved, ensuring that all trajectories point inwards, as proved by Theorem B.1. Consequently, according to the Poincaré–Bendixson Theorem [37], a stable limit cycle which is globally stable exists within this region. This is shown in figure 8a by a closed path highlighted in the same green colour, attracting nearby trajectories towards it. Choosing $a_{1}=4$ and $a_{5}=1.5$ , we create a phase plane diagram and observe the solution for t∈[0,200] to validate the conclusions drawn from the bifurcation analysis detailed in figure 11. The results are summarized in figure 8. One of the questions we want to explore is what is the effect of adding diffusion to this stable limit cycle, does it get stabilized or not? This will be numerically explored in §2.4.

**Region IV.** The region coloured grey in figure 11 is characterized by bistable dynamics. There are three uniform steady states $P_{4}$ , $P_{5}$ and $P_{6}$ . $P_{4}$ and $P_{6}$ are L.A.S and are separated by a saddle point $P_{5}$ . We generate the phase plane diagram using $a_{1}=4.3$ , and $a_{5}=2.1$ , together with two sets of initial conditions (0.6,5) and (2.1,5.5) . The results shown in figure 10 validate the bifurcation analysis results in figure 11. The character of the uniform steady states based on numerically computed eigenvalues summarized in table 5 are L.A.S ( $P_{4}$ ), saddle point ( $P_{5}$ ) and L.A.S ( $P_{6}$ ). The sign patterns of the Jacobian matrices evaluated at the stable uniform steady states $P_{4}$ and $P_{6}$ are given as $\left[\begin{array}{cc} + & -\\ + & - \end{array}\right]$ and $\left[\begin{array}{cc} - & -\\ + & - \end{array}\right]$ , respectively. The only uniform steady state that can admit diffusion-driven instability is $P_{4}$ . In figure 10a, we show the set of initial conditions in the purple and gold regions that lead system (2.3) to converge to the stable uniform steady states $P_{4}$ and $P_{6}$ , respectively.

**Table 5.** A summary of the linear stability analysis of the uniform steady states corresponding to regions I–V of figure 11.

region and figure	uniform steady state	Jacobian matrix	eigenvalues
I single steady state $a_{1}=0.2,a_{5}=2$ figure 6	$P_{1}=(0.024877,0.17634)$	$\left[\begin{array}{cc} -0.0250 & -0.0653\\ 0.9234 & -0.12752 \end{array}\right]$	−0.07626+0.2404i −0.07626−0.2404i
II single Turing-type steady state $a_{1}=2$ , $a_{5}=1$ figure 7b	$P_{2}=$ (0.1270, 0.9864)	$\left[\begin{array}{cc} 0.0483 & -0.1077\\ 0.9147 & -0.1057 \end{array}\right]$	−0.0287+0.3043i −0.0287−0.3043i
III single unstable steady state $a_{1}=4,a_{5}=1.5$ figure 8	$P_{3}=$ (0.34649, 3.2612)	$\left[\begin{array}{cc} 0.0745 & -0.0548\\ 0.8747 & -0.0737 \end{array}\right]$	0.0004+0.2059i 0.0004−0.2059i
IV three steady states two stable and a saddle $a_{1}=4.3$ , $a_{5}=2.1$ figure 10	$P_{4}=$ (0.49027, 4.9289) $P_{5}=$ (2.1613, 7.32) $P_{6}=$ (4.2307, 7.4191)	$\left[\begin{array}{cc} 0.0545 & -0.0367\\ 0.8089 & -0.0780 \end{array}\right]$ $\left[\begin{array}{cc} 0.08127 & -0.0608\\ 0.2285 & -2.1515 \end{array}\right]$ $\left[\begin{array}{cc} -0.6002 & -0.0709\\ 0.1175 & 5.4569 \end{array}\right]$	−0.1176+0.1589i −0.1176−0.1589i 0.0750 −2.1453 −0.6020 −5.4552
V three steady states one unstable a saddle and one stable $a_{1}=5$ , $a_{5}=1.5$ figure 9	$P_{7}=$ (0.43116, 4.2716) $P_{8}=$ (2.1663, 7.3206) $P_{9}=$ (4.2787, 7.4201)	$\left[\begin{array}{cc} 0.0906 & -0.0499\\ 0.8416 & -0.0719 \end{array}\right]$ $\left[\begin{array}{cc} 0.1171 & -0.0708\\ 0.2280 & -2.1593 \end{array}\right]$ $\left[\begin{array}{cc} -0.9506 & -0.0783\\ 0.1162 & -5.5347 \end{array}\right]$	0.0093+0.1881i 0.0093−0.1881i 0.110 −2.1522 −0.9526 −5.5328

**Region V.** The region coloured yellow is characterized by three uniform steady states; namely; $P_{7}$ , $P_{8}$ and $P_{9}$ . Fixing $a_{1}=5$ and $a_{5}=1.5$ , we compute the uniform steady states as shown in figure 9. They are; unstable $\left(P_{7}\right)$ , a saddle point $\left(P_{8}\right)$ and L.A.S $\left(P_{9}\right)$ . We generate a phase plane diagram and the solution for t∈[0,200] to validate the bifurcation results shown in figure 11. The results are illustrated in figure 9. The saddle $P_{8}$ separates the uniform steady states $P_{7}$ and $P_{9}$ so that the trajectories migrate to the steady states, creating trapping regions. By the Poincaré–Bendixson Theorem [37] the uniform steady state $P_{9}$ is L.A.S. and there is a limit cycle which is L.A.S. around the uniform steady state $P_{7}$ . In figure 9a, we show the basin of attraction for the initial conditions within the purple region leading system (2.3) towards convergence to the stable limit cycle marked by the green circle, and the basin of attraction in gold for initial conditions indicating attraction towards the stable uniform steady state $P_{9}$ . We summarize the numerical results in table 5 highlighting the parameter values for $a_{1}$ and $a_{5}$ used to compute the uniform steady states, the Jacobian matrices and their eigenvalues. In §2.4, we will explore, using numerical simulations, the effects of adding diffusion to the temporal ODE system with a stable limit cycle.

### 2.3. Mathematical analysis of the spatio-temporal model

#### 2.3.1. Exploring conditions for diffusion-driven instability

In this section, we will investigate the potential existence of spatial patterns within the system described by system (2.1). These patterns manifest as a consequence of a stable uniform steady state in the absence of diffusion, which becomes unstable when subjected to perturbations in the presence of diffusion. This phenomenon is commonly known as DDI or Turing instability [27,36,38,39]. This instability explains how small local fluctuations in an otherwise well-mixed system of autocatalytic and inhibitory diffusing biochemical species can become unstable owing to diffusion. As a result, spatial patterns emerge when the domain size exceeds a certain critical threshold. The key point is that diffusion in combination with specific reaction kinetics leads to spatial organization or patterns, especially as domain size plays a crucial role in determining stability or instability [27,40–43]. Extensive studies on such patterns in two-component systems have been conducted [36,38,44,45]. Sarfaraz and Madzvamuse delved into bifurcation analysis with Schnakenberg reaction-diffusion kinetics [46]. Their analysis established a relationship between the domain size, reaction-diffusion rates, and the type of DDI. The emergence of spatial patterns may be driven by environmental factors, where local or global variability in driving variables imposes a spatial structure on species concentration [47]. Alternatively, spatial patterns can arise and persist in homogeneous settings owing to domain and diffusion of interacting species [36,48]. Konow *et al*. highlight how chemical reaction-diffusion systems, particularly the chlorite-iodide-malonic acid reaction, have provided a valuable platform for studying and verifying Turing patterns. These chemical systems have been instrumental in simulating and observing behaviours analogous to biological patterns, such as stripes and spots, as seen in animal skins [42]. By combining theoretical analysis with numerical simulations, Van Gorder demonstrates how spatial heterogeneity introduces mode mixing, where different Turing modes grow at similar rates and contribute to the formation of complex patterns. This approach provides new insights into the role of spatial heterogeneity in biological, chemical and physical systems, offering ways to manipulate and understand pattern formation in systems with varying spatial properties [43]. Kondo *et al*. provides a detailed review of how Turing's reaction-diffusion theory applies to zebrafish skin patterns, which serve as a model system for biological pattern formation. While classical reaction-diffusion systems involve chemical concentration changes, zebrafish patterns are established through interactions between pigment cells (melanophores, xanthophores and iridophores) via direct cell contact and cell protrusions, rather than molecular diffusion [41].

The mathematical conditions for DDI are established by initially slightly perturbing the stable uniform steady state. Subsequently, linear stability analysis is performed to determine whether these small perturbations will grow or decay over time [36,47]. Please see appendix C on the derivation of the necessary conditions for Turing DDI.

#### 2.3.2. Turing parameter spaces

To proceed, next we explore the parameter spaces governing DDI, by using the reaction kinetics of our model system (2.1). To do this, we search for parameter values that satisfy the Turing conditions described in inequalities [Disp-formula A2-E11](C 11) and (C 12), ensuring a stable uniform steady state. Our method involves adjusting pairwise parameter values and the diffusion coefficient d , while keeping all others constant, and then plotting the region that fulfills the conditions in (C 11) and (C 12). This helps us identify where exactly within the parameter space a given pair of parameters need to be for Turing instability to occur. For illustrative purposes, we show some selected parameter pairs and corresponding figure references in table 3. It is essential to note that the parameter d represents the ratio of the diffusion rates of u to v . The key principle underlying Turing instability conditions on stationary domains is that the inhibitor should diffuse considerably faster than the activator. This phenomenon leads to pattern formation through *short-range activation* and *long-range inhibition* [38,49]. Additionally, it is important to understand that the extent of the Turing parameter space is directly proportional to d . To demonstrate this, we pick $a_{3}=0.1$ and $a_{7}=16$ from the Turing space $\left(a_{3},a_{7}\right)$ , and use the other parameters taken from set 1 as listed in table 6 to calculate the uniform steady state $\left(u^{*},v^{*}\right)$ for the system (2.1). The homogeneous steady state, determined through numerical computation, is found to be (2.3176,4.4414) . The partial derivatives of f and g evaluated at this homogeneous steady state are denoted as:


$$\begin{array}{rl} f_{u} & =\frac{-a_{1}(a_{3}(a_{4}v(2u-a_{2})+u)-a_{4}v(u-a_{2}{)}^{2})}{(a_{2}+a_{3}-u{)}^{2}(a_{4}v+u{)}^{2}}-\frac{a_{5}}{(a_{5}+u{)}^{2}}=0.0798,\\ f_{v} & =-\frac{a_{1}a_{4}u(a_{2}-u)}{(a_{2}+a_{3}-u)(a_{4}v+u{)}^{2}}=-0.1028,\\ g_{u} & =\frac{a_{6}-v}{a_{6}+a_{7}-v}=0.1605,\;\text{and}\;g_{v}=-\frac{a_{7}v}{(a_{6}+a_{7}-v{)}^{2}}-\frac{a_{8}}{(a_{8}+v{)}^{2}}=-0.1547. \end{array}$$

The Jacobian matrix evaluated at $(u^{*},v^{*})=\left(2.3176,4.4414\right)$ is thus given by:


(2.4) $$\begin{array}{lrr} & J_{\left(u^{*},v^{*}\right)}={\left(\begin{array}{cc} f_{u} & f_{v}\\ g_{u} & g_{v} \end{array}\right)}_{\left(u^{*},v^{*}\right)}=\left(\begin{array}{cc} 0.07980 & -0.1028\\ 0.16048 & -0.15468 \end{array}\right), & \end{array}$$

of which the trace T=−0.07488<0 and the determinant D=0.0041388>0. This guarantees the fulfillment of the first two conditions for DDI stated in (C 11) and (C 12). For these selected model parameters, it follows from (C 17) that the critical diffusion coefficient $d_{c}$ is given by $d_{c}=0.642$ or $d_{c}=5.84$ . It is noteworthy to emphasize that equation (C 17) dictates that the diffusion coefficient ratio d must exceed 1. Therefore, we take the value of the critical diffusion coefficient ratio $d_{c}=5.84$ . Having determined the critical diffusion coefficient, $d_{c}$ , we proceeded to isolate possible wave numbers with varying γ and fixed d=10 . For the interested reader, please see appendix D for details on mode isolation.

In the next section, we simulate system (2.1) together with reaction kinetics (2.2) to illustrate the effect of adding diffusion to the ODE system in different regions. For illustrative purposes unless otherwise stated, we will only present solutions corresponding to u(x,t) .

### 2.4. Numerical simulations of the reaction-diffusion system

Given that the temporal behaviour of the model is characterized by the five regions: regions I to V, we want to understand how adding diffusion will affect the temporal system in the case where: (i) there is a unique stable uniform steady state (non-Turing uniform steady state); (ii) unique stable uniform steady state (leading to diffusion-driven instability); (iii) unique unstable uniform steady state (leading to temporal periodic solutions); (iv) three uniform steady states: two stable separated by a saddle (bistability); and (v) three uniform steady states; stable, a saddle and unstable (exhibiting coexistence of a stable limit cycle and a stable uniform steady state). We simulate in 1-space dimension the numerical solutions corresponding to the reaction-diffusion system (2.1) using *pdepe* in MATLAB to illustrate the effects of adding diffusion to the temporal system in all five regions shown in figure 11. *pdepe* is a MATLAB toolbox that offers a convenient and powerful approach to solving PDEs in 1-spatial dimension, incorporating both initial and boundary conditions. It proves especially beneficial for solving parabolic and elliptic PDEs, which are frequently encountered in various fields like physics, engineering and biology. It is based on the method of lines, where the spatial variable is discretized and the ensuing system of ODEs is then solved [50]. We note that similar numerical solutions are obtained using an advanced numerical method known as the finite element method (FEM). The FEM is implemented in the open source software package known as *FeNiCs* [51]. Having the two different numerical methods allows us to validate the computational solvers and their convergence. In this study, we will only show the results obtained using the *pdepe* solver.

#### 2.4.1. Numerical simulations in region I

**Figure 12.**
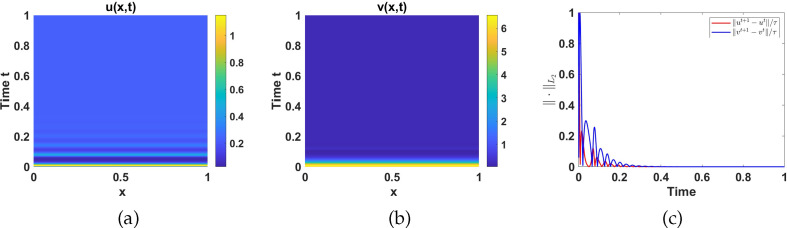
Plots of: (a) u(x,t) , and (b) v(x,t) numerical solutions, and (c) the $L_{2}$ -norm of the discrete time-derivative of u(x,t) and v(x,t) at different time points. Parameters are chosen from region I while the rest are as listed in table 6 with d=10 and γ=500 .

Region I is characterized by a unique stable uniform steady state (spiral) denoted $P_{1}$ . For numerical simulations, we set $a_{1}=0.2$ and $a_{5}=2$ from region I in figure 11, with d=10 , γ=500 and set 2 parameters as listed in table 6. See figure 12 for the numerical simulation results corresponding to system (2.1) in region I.

As shown in figure 12a,b, for any initial conditions, the system converges to the uniform steady state (0.024877,0.17634) , and therefore adding diffusion to the temporal model does not induce instability. This is confirmed by the $L_{2}$ -norm of the discrete time-derivative as shown in figure 12c, which decays for all time, indicating the stability of the uniform steady state (0.024877,0.17634) and the absence of spatial patterns.

#### 2.4.2. Numerical simulations in region II

Region II is characterized by a stable spiral uniform steady state, as shown in figure 11. The corresponding Jacobian matrix at this uniform steady state has the sign matrix, $\left(\begin{array}{cc} + & -\\ + & - \end{array}\right)$ , indicating the potential for Turing pattern formation, since the first two conditions of Turing instability are satisfied [36].

For numerical simulations in this region, we first follow the standard Turing analysis as described in §2.2 to generate Turing spaces (see figure 4) with different pairwise parameter combinations. In table 7, we show the different parameter spaces when the diffusion coefficient d is varied. Following this, we introduce small random perturbations to the uniform steady state and proceed to simulate the nonlinear reaction-diffusion system (2.1). To exemplify this approach, let us focus on the first mode, where k=π , corresponding to γ=500 . The corresponding eigenfunction is given by $\Phi_{1}(x)=\cos\;\pi x$ . We take initial conditions as random perturbations around the uniform steady state [38,52] in the form:


$$u_{0}=u^{*}+\epsilon \times \text{rand}\times \cos(x\pi ),\;\text{and}\;v_{0}=v^{*}+\epsilon \times \text{rand}\times \cos(x\pi ).$$

**Figure 13.**
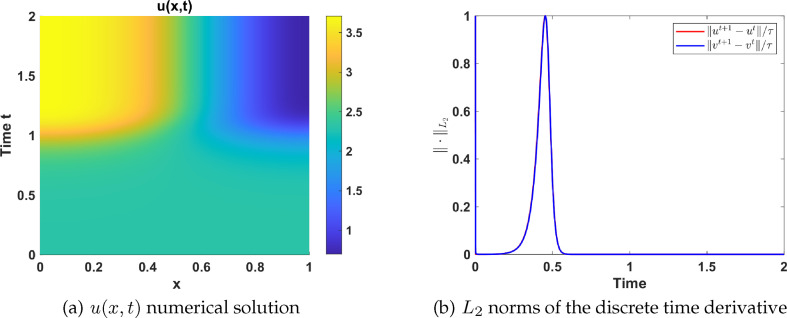
Plot of: (a) the contours of u(x,t) numerical solutions and (b) the $L_{2}$ norms of the discrete time-derivatives of u(x,t) and v(x,t) . The parameter values are chosen from region II while the rest are listed in table 6 with d=10 and γ=500 . The numerical solutions qualitatively mirror the profile of the eigenfunction.

With parameter values $a_{3}=0.1$ and $a_{7}=16$ selected from the $\left(a_{3},\;a_{7}\right)$ parameter space shown in figure 4b and others from fixed set 2 as listed in table 6. We find the uniform stead state, $\left(u^{*},v^{*}\right)$ = (2.3176,4.4414) and take $\epsilon =10^{-3}$ . We plot the numerical solution u(x,t), corresponding to the reaction-diffusion system (2.1) with the perturbed initial conditions above and no flux boundary conditions. The results are shown in figure 13. The convergence in the $L_{2}$ norm of the discrete time derivatives of the solutions u and v is shown in figure 13c.

In all subsequent simulations, we take $a_{3}=0.1$ , $a_{7}=16$ and fix all the other parameters in set 2 as listed in table 6 including d=10 and vary γ . For γ=1000 , the linearized system has three eigenfunctions $\Phi_{1}x=cos\;\pi x$ and $\Phi_{2}x=cos\;2\pi x$ originating from the two modes, one with k=π and the other with k=2π and the linear combination of the two, that is, $\Phi_{3}x=a_{1}cos\;\pi x\;\;+\;\;a_{2}cos\;2\pi x$ . From these, we generate three distinct initial conditions given as:


(2.5) $$\begin{array}{lrlr} & & \left\{ \begin{array}{c} u_{0}(x)=u^{*}+\epsilon \times \text{rand}\times \cos(x\pi ),\\ v_{0}(x)=v^{*}+\epsilon \times \text{rand}\times \cos(x\pi ), \end{array}\right. & \end{array}$$

(2.6) $$\begin{array}{lrlr} & & \left\{ \begin{array}{c} u_{0}(x)=u^{*}+\epsilon \times \text{rand}\times \cos(2x\pi ),\\ v_{0}(x)=v^{*}+\epsilon \times \text{rand}\times \cos(2x\pi ), \end{array}\right. & \end{array}$$

and


(2.7){u0(x)=u∗+ϵ×rand×[cos⁡(xπ)+cos⁡(2xπ)],v0=v∗+ϵ×rand×[cos⁡(xπ)+cos⁡(2xπ)].

**Figure 14.**
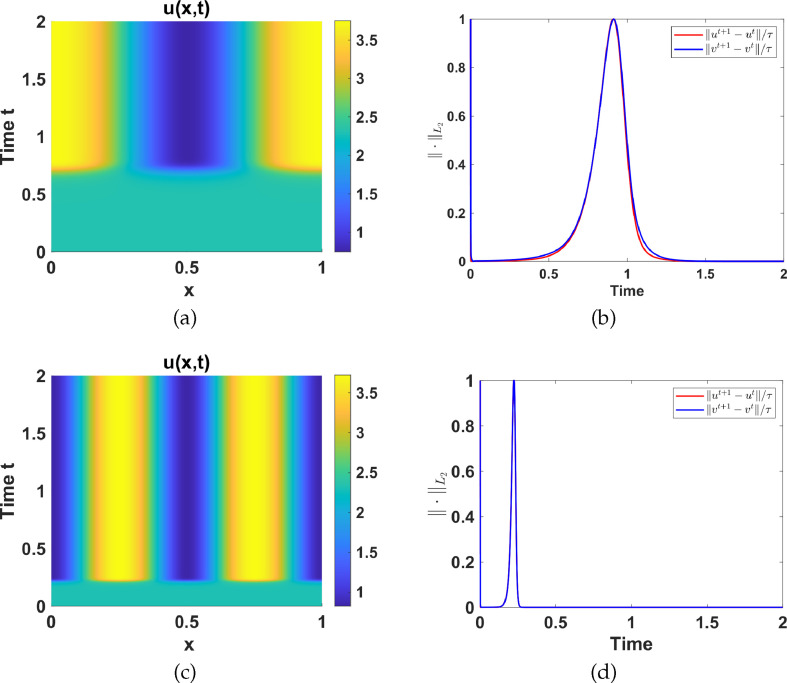
Numerical simulation results corresponding to system (2.1) with parameter values in region II, *d* = 10 and varying *γ*.(a) u(x,t) , numerical solution and (b) the $L_{2}$ norms of the discrete time-derivative of u(x,t) and v(x,t) , with γ=1000 , while (c) and (d) are respectively the contour plot of u(x,t) and the $L_{2}$ norms of the discrete time-derivative of u(x,t) and v(x,t) , when γ=5000 . The profiles of numerical solutions qualitatively reproduce the profiles of the eigenfunctions as predicted by the linear stability theory.

Owing to the existence of multiple excitable wave modes, for the same parameter values, system (2.1) admits different solutions that depend on the choice of initial conditions, which act as the basin of attraction closet to the wave number to be excited. For illustrative purposes, we only use the initial condition (2.6) to simulate system (2.1). To also illustrate the effect of varying γ on the numerical simulation results, we set γ=5000 and simulate system (2.1) with d=10 and other parameter values fixed as in table 6. With this γ , the number of strips increases (see figure 14).

#### 2.4.3. Numerical simulations in Region III

**Figure 15.**
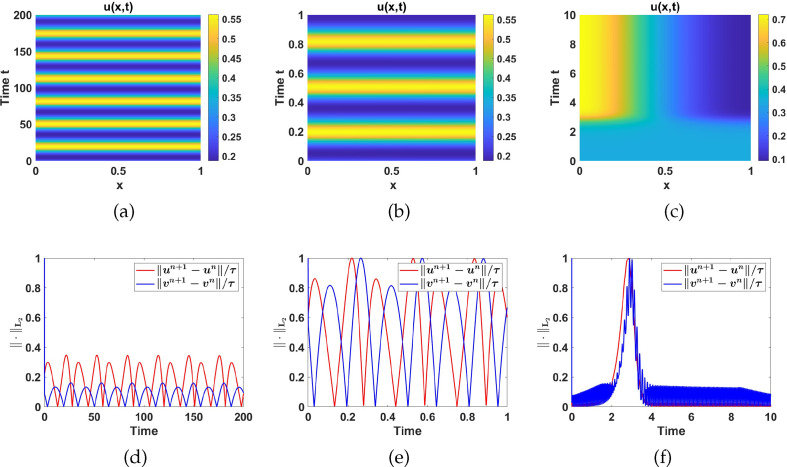
Numerical simulation results corresponding to system (2.1) with parameter values in region III and *d* = 50.(a)–(c) Contour plots of u(x,t) for γ=1 , γ=100 and γ=250 respectively, and (d)–(e) the respective $L_{2}$ -norm of the discrete time-derivative of u(x,t) and v(x,t) .

Region III is characterized by a unique unstable uniform steady state surrounded by a stable limit cycle as shown in figure 8. To illustrate the effect of adding diffusion to the temporal model in this region, we set $a_{1}=4$ and $a_{5}=1.5$ and fix the other set 2 parameter values as listed in table 6. See figure 15 for numerical simulation results. For illustrative purposes, we show that for γ=1 and γ=100 in figure 15 the limit cycle is not destabilized.

**Figure 16.**
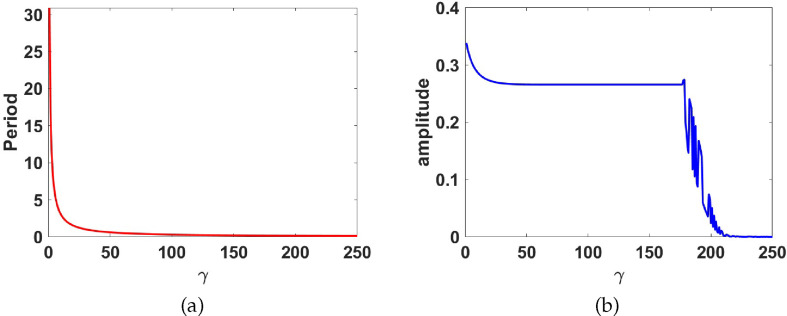
Plots of (a) the period and (b) amplitude, against γ respectively, corresponding to the limit cycle in region III, with d=50 .

To show the effect of varying γ on the limit cycle in region III, we plot the period and the amplitude of the limit cycle as functions of γ (see figure 16). The limit cycle remains stable when diffusion is added for 1≤γ≤177.5 . However, for γ>177.5 , the limit cycle begins to destabilize, leading to the emergence of spatial patterns. This phenomena has been observed in other studies of this nature, see [53]. For γ=1 , the limit cycle has a period of 30.901. As γ increases, the period rapidly decreases and remains fairly constant for 25<γ<177.5 , after which the period rapidly decreases to zero for γ>177.5 (see figure 16a). The same observation is made with the amplitude as shown in figure 16b. Consequently, the introduction of diffusion leads to the destabilization of the limit cycle for certain values of d and γ .

#### 2.4.4. Numerical simulations in region IV

**Figure 17.**
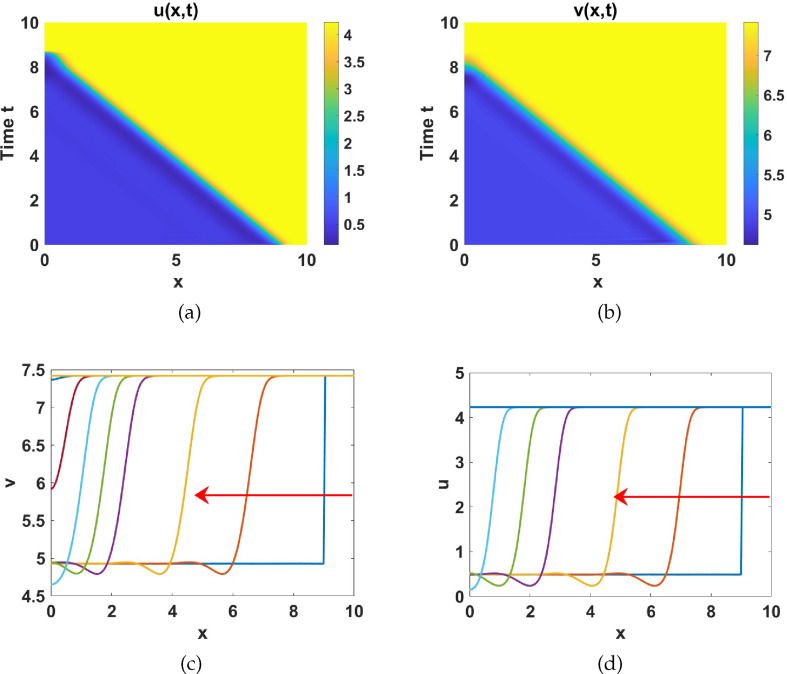
Plots of: (a) u(x,t) , and (b) v(x,t) solutions exhibiting travelling wave fronts and spatial profiles of (c) u(x,t) and (d) v(x,t) at different time points. Parameters are chosen from region IV while the rest are listed in table 6 together with d=50 and γ=200 .


**Figure 18.**
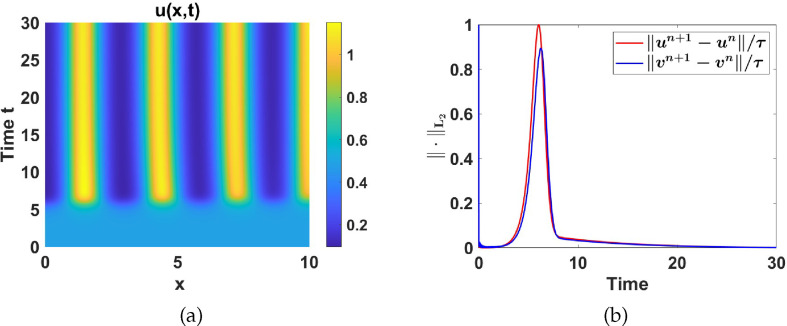
Plots of: (a) contour plot of u(x,t) , exhibiting the formation of spatially inhomogeneous patterns. (b) Plot of the $L_{2}$ -norm of the discrete time derivatives of u(x,t) and v(x,t) . Parameters are chosen from region IV while the rest are listed in table 6 together with d=50 , γ=200 and L=10 .

Here we explore the effect of adding diffusion to the ODE system (2.3) on the dynamical region IV, which is characterized by three uniform steady states: two stable equilibria, and a saddle as shown in figure 11. We present numerical simulation results using parameter values extracted from set 2 as listed in table 6, and let $a_{1}=4.3$ and $a_{5}=2.1$ . Initial conditions are prescribed as:


$$\begin{array}{r} \begin{array}{r} (u_{0}(x),v_{0}(x){)}^{T}=\left\{ \begin{array}{l} \left\{ \begin{array}{l} u_{P_{4}}^{*}(x)+\epsilon \times \text{rand}\times \cos(x\pi /L_{x})\\ v_{P_{4}}^{*}(x)+\epsilon \times \text{rand}\times \cos(x\pi /L_{x}), \end{array}\right.\;x\leq 9\\ \left\{ \begin{array}{l} u_{P_{6}}^{*}(x)+\epsilon \times \text{rand}\times \cos(x\pi /L_{x})\\ v_{P_{6}}^{*}(x)+\epsilon \times \text{rand}\times \cos(x\pi /L_{x}), \end{array}\right.\;\text{elsewhere} \end{array}\right. \end{array} \end{array}$$

in a domain of length $L_{x}=10$ . We use here an enlarged domain to see how the wave fronts travel through the domain. In figure 17, we observe how diffusion leads to the emergence of a traveling wave front from the uniform steady $P_{4}$ to $P_{6}$ . This phenomena has been observed analytically and recently published [53]. Consequently, we identify travelling wave fronts that progress from left to right, as shown in figure 17. We proceed to investigate the effect of diffusion to system (2.2) using the initial condition:

$$\begin{array}{r} (u_{0}(x),v_{0}(x){)}^{T}=\left\{ \begin{array}{c} u_{P_{4}}^{*}(x)+\epsilon \times \text{rand}\times \cos(x\pi )\\ v_{P_{4}}^{*}(x)+\epsilon \times \text{rand}\times \cos(x\pi ), \end{array}\right. \end{array}$$

and the same domain length $L_{x}=10$ . The numerical results shown in figure 18 highlight the interplay between diffusion and reaction terms in pattern formation and front dynamics. This behaviour is consistent with findings from studies such as Champneys *et al*. [54] and Burke & Knobloch [55], which emphasized the role of bistability and homoclinic snaking in systems with subcritical and supercritical regimes. In particular, bistability arises in pinning regions where stationary fronts stabilize localized structures. While often associated with subcritical Turing bifurcations, transitions to supercritical regimes can sustain bistability under specific nonlinear interactions or parameter configurations. These insights align with the observed dynamics here, where diffusion mediates spatial patterning and front stabilization in a reaction-diffusion system.

#### 2.4.5. Numerical simulations in region V

**Figure 19.**
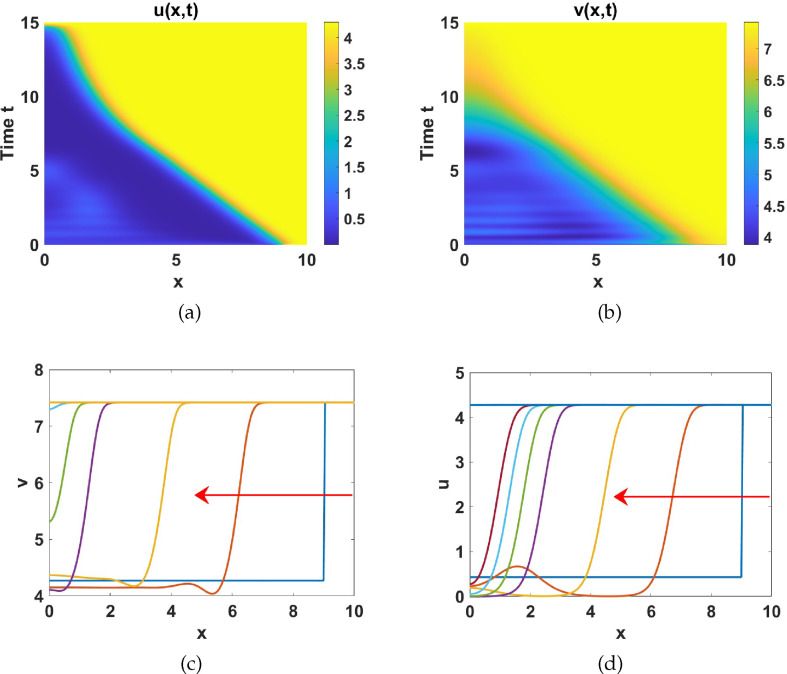
Plots of: (a) u(x,t) , and (b) v(x,t) solutions exhibiting travelling wave fronts. (c) –(d) Snapshots of the spatial profiles of u(x,t) and v(x,t) at different time points. Parameters are chosen from region V while the rest are listed in table 6 together with d=50 , γ=200 .

Region V is characterized by three coexisting uniform steady states: $P_{7}$ (unstable), $P_{8}$ (saddle) and $P_{9}$ (stable). There is a locally asymptotically stable limit cycle around the uniform steady state $P_{7}$ as shown in figure 9. To show the effects of diffusion in this dynamic region, we prescribe the initial condition as:


$$\begin{array}{r} \begin{array}{r} (u_{0}(x),v_{0}(x){)}^{T}=\left\{ \begin{array}{l} \left\{ \begin{array}{l} u_{P_{7}}^{*}(x)+\epsilon \times \text{rand}\times \cos(x\pi /L_{x})\\ v_{P_{7}}^{*}(x)+\epsilon \times \text{rand}\times \cos(x\pi /L_{x}), \end{array}\right.\;x\leq 9\\ \left\{ \begin{array}{l} u_{P_{9}}^{*}(x)+\epsilon \times \text{rand}\times \cos(x\pi /L_{x})\\ v_{P_{9}}^{*}(x)+\epsilon \times \text{rand}\times \cos(x\pi /L_{x}), \end{array}\right.\;\text{elsewhere} \end{array}\right., \end{array} \end{array}$$

with $a_{1}=5$ and $a_{5}=1.5$ and fix the other parameters as listed in table 6 (set 2). Simulating the system (2.1) under these conditions results in travelling wave fronts from the locally unstable uniform steady state $P_{7}$ to the locally stable steady state $P_{9}$ as shown in figure 19.

## 3. Conclusion and future studies

Mathematical and computational models have proved useful tools to help theoretical scientists, clinician and experimentalists gain understanding of biological processes and to make experimentally verifiable predictions [4,21,23,56]. With increasing availability of data-driven mathematical models, detailed mathematical and numerical analysis are needed to understand their dynamics. Kamps *et al*. [21] proposed one of these models to describe the interaction between GEF-Rho-myosin linked to cell contraction. Therefore, in this study, we focused on the detailed mathematical analysis of a reaction-diffusion system of activator-inhibitor type, with experimentally driven reaction kinetics. This reaction kinetics for this reaction-diffusion system were proposed in previous studies [21,23]. Our aim in this study was to unravel the effects of diffusion on the temporal dynamical system characterized by different temporal behaviour; ranging from a unique uniform steady state (stable or unstable) and the coexistence of multiple stable uniform steady states (bistability), or the coexistence of stable uniform steady states with limit cycles (bistability).

The analysis of this model followed a twofold approach. (i) The spatially homogeneous model was analysed using linear stability theory, phase plane analysis and numerical bifurcation analysis to characterize the model in terms of different temporal dynamical behaviour, and numerical simulations used to illustrate the results. (ii) The full reaction-diffusion system was analysed in terms of Turing analysis and numerical simulations to study the effect of adding diffusion to the temporal model for different parameter regimes characterized by different temporal dynamics. Using numerical bifurcation analysis, we first classified the ODE model, identifying two different sets of parameters that gave rise to different dynamics by varying the bifurcation parameter ( $a_{1}$ ). For the first set of parameters (set 1), the ODE model exhibits two dynamical regimes; transitioning from stable to oscillatory and back to stable as the bifurcation parameter ( $a_{1}$ ) changes. For small values of $a_{1}$ , the model exhibits stable dynamics. As $a_{1}$ increases, for intermediate values, the system is characterized by periodic solutions arising from a Hopf bifurcation, and for large values, the system returns to stable dynamics.

On the other hand, with the set 2 parameter values, the ODE model exhibits up to five distinct dynamical regimes. As $a_{1}$ increases, the ODE model changes from having a unique uniform steady state (asymptotically stable, divided into non-Turing- and Turing-type uniform steady states; and an unstable steady state surrounded by a stable limit cycle). An increase in $a_{1}$ leads to multiple uniform steady states; from having two stable uniform steady states separated by a saddle point; to stable and unstable uniform steady states separated by a saddle point. The dynamical regimes are summarized as follows:

- asymptotically stable non-Turing uniform steady states for values of $a_{1}$ such that $0<a_{1}<0.1.03106$ and $a_{1}>6.6$ ;
- asymptotically stable Turing type uniform steady state for $1.03106<a_{1}<3.965$ ;
- stable limit cycle for $3.965<a_{1}<4.174$ ;
- three coexisting uniform steady states: one stable, one saddle and one unstable for $4.174<a_{1}<6.293$ ; and
- bistability for $6.293<a_{1}<6.6$ .

We further studied the effect of adding diffusion to the ODE model in all five identified dynamical regimes, and these are summarized as follows. Addition of spatial variations to the temporal ODE system within each of the dynamical regions reveals the following dynamics:

- diffusion has no effect in region I;
- in region II, addition of diffusion gives rise to spatial patterns (diffusion-driven instability);
- in region III, diffusion destablizes the stable limit cycle giving rise to spatial patterns;
- in region IV, diffusion destabilizes the lower uniform steady state leading to the formation of spatial patterns; and
- in region IV and V, the diffusion gives rise to travelling wave fronts.

## Figures and Tables

**Figure 20.**
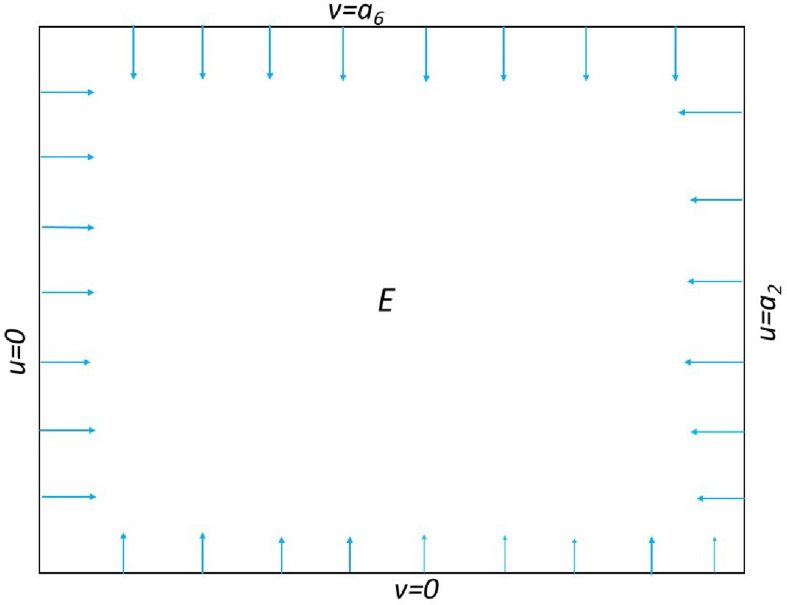
A schematic representation showing the flow of solutions when approaching the boundaries of the invariant set E .

**Figure 21.**
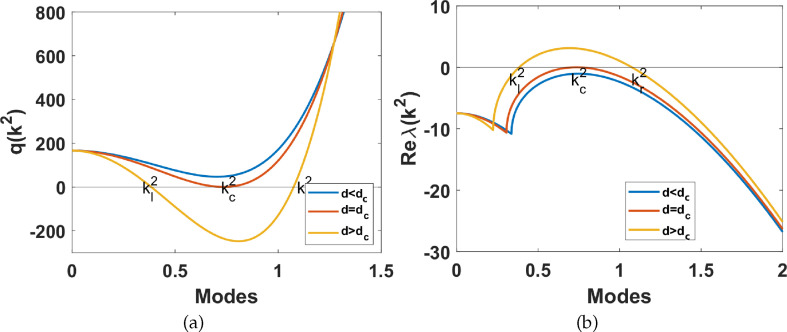
Plots illustrating the interval $k_{l}^{2}<k^{2}<k_{r}^{2}$ in which (a) $q(k^{2})<0$ and (b) Re $\lambda (k^{2})>0$ hold for $d<d_{c}$ , $d=d_{c}$ , and $d>d_{c}$ . Here, $d_{c}=5.84$ , γ=200 , $a_{7}=16$ , in addition to the other parameters from set 2 as listed in table 6.

**Figure 22.**
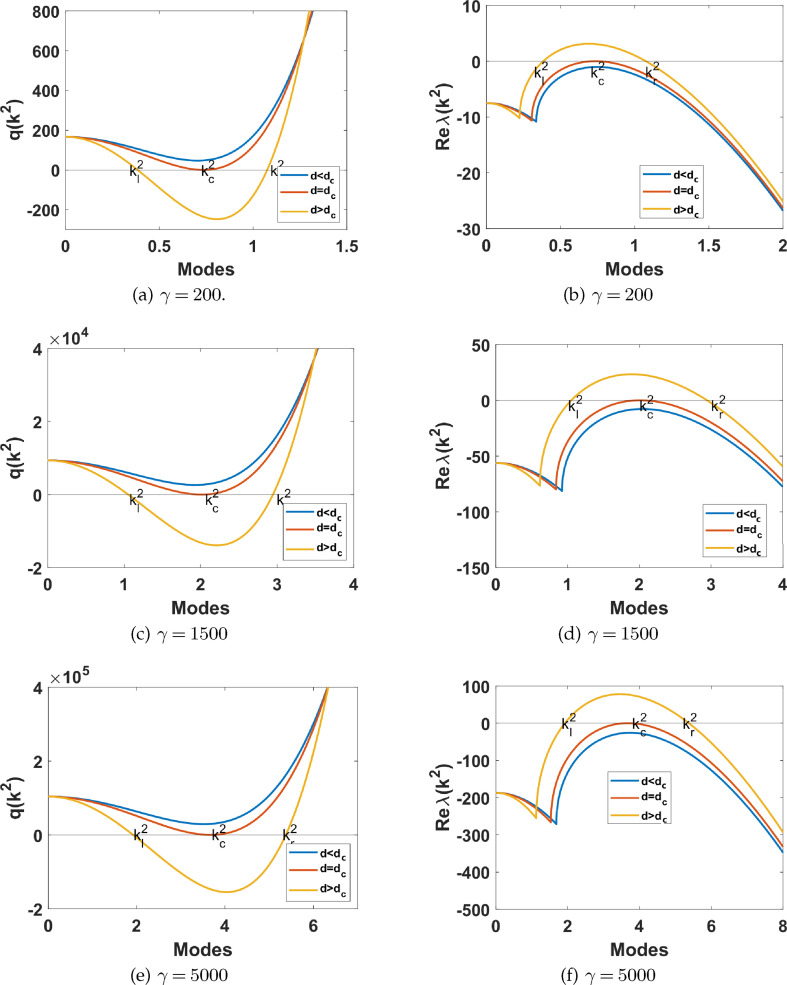
Plots for $q(k^{2})$ and Re $\lambda (k^{2})$ showing the excited modes between $k_{l}^{2}$ and $k_{r}^{2}$ . (a) – (b): one excited mode, with γ=200 . (c) –(d): one excited mode, γ=1500 . (e) – (f): three excited modes, γ=5000 . See table 7 for the summary of excited modes.

## Data Availability

The current manuscript includes all the data supporting the findings of this study.

## Appendix A. Non-dimensionalization

To non-dimensionalize system (2.1), we set $R_{a}=U\hat{u},\;\;M_{a}=V\hat{\upsilon },\;\;x=L_{x}\hat{x}\;\;\;and\;\;\;t=T\hat{t}$ where $L_{x}$ is the length scale in one-dimension. Substituting these into system (2.1) with reaction kinetics (2.1) and defining

$$\begin{array}{rl} a_{1} & =\frac{k_{1}G_{T}}{k_{2}},\;a_{2}=\frac{R_{T}k_{5}}{k_{6}},\;a_{3}=\frac{K_{m_{1}}k_{5}}{k_{6}},\;a_{4}=\frac{k_{4}k_{6}}{k_{2}k_{3}},\\ a_{5} & =\frac{K_{m_{2}}k_{5}}{k_{6}},\;a_{6}=\frac{M_{T}k_{2}k_{5}}{k_{6}^{2}},\;a_{7}=\frac{K_{m_{3}}k_{2}k_{5}}{k_{6}^{2}},\\ a_{8} & =\frac{k_{m_{4}}k_{2}k_{5}}{k_{6}^{2}},\;d=\frac{D_{myo}}{D_{rho}},\;\gamma =\frac{L_{x}^{2}k_{2}k_{5}}{k_{6}D_{rho}}, \end{array}$$

where $U=\frac{k_{6}}{k_{5}},V=\frac{k_{6}^{2}}{k_{2}k_{5}}\;and\;T=\frac{L_{x}^{2}}{D_{rho}}$ we obtain the dimensionless system:

(A 1)
$$\begin{array}{r} \left\{ \begin{array}{l} \left\{ \begin{array}{l} \frac{\partial u}{\partial t}=\gamma f(u,v)+\nabla^{2}u,\\ \;\;\;\;\;\;\;\;\;\;\;\;\;\;\;\;\;\;\;\;\;\;\;\;\;\;\;\;\;\;\;\;\;\;\;\;\;\;\;\;\;\;\;\;\;\;\;\;x\in \Omega ,\;t>0,\\ \frac{\partial v}{\partial t}=\gamma g(u,v)+d\nabla^{2}v, \end{array}\right.\\ -(n\cdot \nabla u)=0,\;-d(n\cdot \nabla v)=0,\;\;\;x\in \partial \Omega ,\;t>0,\\ u(x,0)=u_{0}(x),\;v(x,0)=v_{0}(x),\;\;\;\;\;\;x\in \Omega ,\;t=0, \end{array}\right. \end{array}$$

where

(A 2)
$$\begin{array}{lcr} & \left\{ \begin{array}{c} f(u,v)=\frac{a_{1}u(a_{2}-u)}{\left(a_{3}+a_{2}-u)(a_{4}v+u\right)}-\frac{u}{a_{5}+u},\\ g(u,v)=\frac{u(a_{6}-v)}{a_{7}+a_{6}-v}-\frac{v}{a_{8}+v}, \end{array}\right. & \end{array}$$

after dropping the ^ (hats) for notational simplicity. In table 6, we can see in the third column that the newly introduced parameters are dimensionless.

**Table 6.** Non-dimensional parameters are expressed in terms of dimensional quantities. (Here, we illustrate that the parameters are unit-free. The parameter values used for numerical analysis are grouped into two sets: set 1, designed to investigate stable and oscillatory dynamics, and set 2, intended to explore stable, oscillatory and bistable dynamics).

non-dimensional parameters	dimensional parameters	dimensional units	parameter values	
	set 1	set 2		
$a_{1}$	$\frac{k_{1}G_{T}}{k_{2}}$	$\frac{s^{-1}U}{Us^{-1}}$	0.8	4.2
$a_{2}$	$\frac{R_{T}k_{5}}{k_{6}}$	$\frac{Us^{-1}}{Us^{-1}}$	1.5	4.5
$a_{3}$	$\frac{K_{m_{1}}k_{5}}{k_{6}}$	$\frac{Us^{-1}}{Us^{-1}}$	0.0765	0.1
$a_{4}$	$\frac{k_{4}k_{6}}{k_{2}k_{3}}$	$\frac{U^{-1}\;s^{-1}Us^{-1}}{U\;s^{-1}U^{-1}\;s^{-1}}$	0.2857	2.1053
$a_{5}$	$\frac{K_{m_{2}}k_{5}}{k_{6}}$	$\frac{U^{-1}\;s^{-1}U\;s^{-1}U^{2}}{U^{2}\;s^{-2}}$	0.075	1.75
$a_{6}$	$\frac{M_{T}k_{2}k_{5}}{k_{6}^{2}}$	$\frac{s^{-1}\;U}{U\;s^{-1}}$	7.5	7.5
$a_{7}$	$\frac{K_{m_{3}}k_{2}k_{5}}{k_{6}^{2}}$	$\frac{U^{2}\;s^{-1}}{U^{2}\;s^{-1}}$	3.75	0.6075
$a_{8}$	$\frac{k_{m_{4}}k_{2}k_{5}}{k_{6}^{2}}$	$\frac{U^{2}\;s^{-2}}{U^{2}\;s^{-2}}$	5.625	7.5
γ	$\frac{L_{x}^{2}k_{2}k_{5}}{k_{6}D_{rho}}$	$\frac{U^{2}Us^{-1}s^{-1}}{Us^{-1}U^{2}s^{-1}}$	varies	varies
d	$\frac{D_{myo}}{D_{rho}}$	$\frac{U^{2}s^{-1}}{U^{2}s^{-1}}$	varies	varies

## Appendix B. Preliminary of mathematical analysis

### B.1. Positive invariance

To verify the well-posedness of system (2.3), we use the following theorem:

**Theorem B.1.** *The set* $E=\left\{(u,v)\in {\mathbb{R}}^{2}:0\leq u\leq a_{2},\;0\leq v\leq a_{6}\right\}$ *is compact, and positively invariant with respect to the flow of system* (2.3).

*Proof of Theorem B.1.* By the Bony–Brezis theorem [57], it is enough to demonstrate that the vector field induced by the system is tangent or enters E . In other words, the vector field does not allow flow outside the region E . Therefore, we check the flow field close to the boundaries u=0 , $u=a_{2}$ , v=0 and $v=a_{6}$ , as illustrated in figure 20. Following the boundary $u=a_{2}$ , it is clear from equation (2.3) that

$$\begin{array}{r} \lim_{u\to a_{2}}\;\frac{du}{dt}=-\frac{a_{2}}{a_{5}+a_{2}}\leq 0, \end{array}$$

therefore, u is decreasing. Hence, the vector field flows into E . In the same way, along the boundary u=0 , we have:

(B 1)
$$\begin{array}{lrr} & \lim_{u\to 0}\;\frac{du}{dt}=0. & \end{array}$$

This means the vector field is tangential to the boundary of E . Consequently, u does not leave the vector field. On the $v=a_{6}$ boundary, it is clear from equation (2.3) that:

(B 2)
$$\begin{array}{r} \lim_{v\to a_{6}}\frac{dv}{dt}=-\frac{a_{6}}{a_{8}+a_{6}}\leq 0, \end{array}$$

hence v is decreasing, and the vector field flows into E . Similarly, along v=0 boundary:

(B 3)
$$\begin{array}{lrr} & \lim_{v\to 0}\;\frac{dv}{dt}=\frac{ua_{6}}{a_{7}+a_{6}}\geq 0, & \end{array}$$

therefore the flow is into E . The system is considered with initial conditions with $0\leq u(t_{0})\leq a_{2}$ and $0\leq v(t_{0})\leq a_{6}$ . Solutions to the system (2.3) considered with non-negative initial conditions exist and are unique, since the vector field $(f,g)\in C^{\infty }(E)$ . Furthermore, the planar system defined in equation (2.3) has bounded solutions, as proved in Theorem B.1.∎

### B.2. Linear stability analysis in the absence of diffusion

The homogeneous uniform steady state (u*,v*) of system (2.3) calculated numerically satisfies

(B 4)
$$\begin{array}{lrr} & f(u^{*},v^{*})=g(u^{*},v^{*})=0, & \end{array}$$

after disregarding γ because it is strictly positive and has no impact on the temporal dynamic of the system. To linearize equation (2.3), about the uniform steady state $\left(u^{*},v^{*}\right)$ , we let

(B 5)
$$\begin{array}{r} u(t)=u^{*}+\epsilon w_{1}(t)\;\;\;\text{and}\;\;\;v(t)=v^{*}+\epsilon w_{2}(t), \end{array}$$

where ϵ≪1 . Substituting into equation (2.3) and expanding the right-hand side in Taylor series about $\left(u^{*},v^{*}\right)$ , we obtain the following:

(B 6)
$$\begin{array}{lrr} & \epsilon \frac{\partial w_{1}}{\partial t}=f(u^{*},v^{*})+\epsilon w_{1}\frac{\partial f}{\partial u}+\epsilon w_{2}\frac{\partial f}{\partial v}+O(\epsilon^{2}), & \end{array}$$

(B 7)
$$\begin{array}{lrr} & \epsilon \frac{\partial w_{2}}{\partial t}=g(u^{*},v^{*})+\epsilon w_{1}\frac{\partial g}{\partial u}+\epsilon w_{2}\frac{\partial g}{\partial v}+O(\epsilon^{2}). & \end{array}$$

Given $f(u^{*},v^{*})=g(u^{*},v^{*})=0,$ we can equate O(ϵ) terms and neglect $O(\epsilon^{2})$ terms, yielding:

(B 8)
$$\begin{array}{r} \frac{\partial w}{\partial t}=Jw, \end{array}$$

in matrix-vector notation, where $w=\left(\begin{array}{c} w_{1}(t)\\ w_{2}(t) \end{array}\right),$ and $J={\left(\begin{array}{cc} f_{u} & f_{v}\\ g_{u} & g_{v} \end{array}\right)}_{\left(u^{*},v^{*}\right)}$ is the Jacobian matrix evaluated at $\left(u^{*},v^{*}\right)$ . The entries of J are the partial derivatives of f(u,v) and g(u,v) with respect to u and v and these are given by:

$$\begin{array}{rl} f_{u}{|}_{\left(u^{*},v^{*}\right)} & =\frac{-a_{1}(a_{3}(a_{4}v^{*}(2u^{*}-a_{2})+u^{*2})-a_{4}v^{*}(u^{*}-a_{2}{)}^{2})}{(a_{2}+a_{3}-u^{*}{)}^{2}(a_{4}v^{*}+u^{*}{)}^{2}}\\ & -\frac{a_{5}}{(a_{5}+u^{*}{)}^{2}},\\ f_{v}{|}_{\left(u^{*},v^{*}\right)} & =-\frac{a_{1}a_{4}u^{*}(a_{2}-u^{*})}{(a_{2}+a_{3}-u^{*})(a_{4}v^{*}+u^{*}{)}^{2}},\\ g_{u}{|}_{\left(u^{*},v^{*}\right)} & =\frac{a_{6}-v^{*}}{a_{6}+a_{7}-v^{*}},\\ g_{v}{|}_{\left(u^{*},v^{*}\right)} & =-\frac{a_{7}v^{*}}{(a_{6}+a_{7}-v^{*}{)}^{2}}-\frac{a_{8}}{(a_{8}+v^{*}{)}^{2}}. \end{array}$$

The solutions of equation (B 8) [Disp-formula A2-E8] are of the form

(B 9)
$$\begin{array}{lrr} & w=c\;\exp(\lambda t), & \end{array}$$

where λ is the eigenvalue. Substituting equation (B 9) into (B 8), we obtain the eigenvalue λ as the zero of

(B 10)
$$\begin{array}{l} |J-\lambda I|=\left|\begin{array}{cc} f_{u}-\lambda & f_{v}\\ g_{u} & g_{v}-\lambda \end{array}\right|=0. \end{array}$$

From equation (B 10) we obtain the characteristic equation

(B 11)
$$\begin{array}{lrr} & \lambda^{2}-(f_{u}+g_{v})\lambda +(f_{u}g_{v}-f_{v}g_{u})=0, & \end{array}$$

with two solutions:

$$\begin{array}{lrlr} & \lambda_{\pm }= & \frac{1}{2}(\{f_{u}+g_{v}\}\pm \sqrt{\left(f_{u}+g_{v}{)}^{2}-4(f_{u}g_{v}-f_{v}g_{u}\right)}) & \\ & = & \frac{1}{2}(Tr(J)\pm \sqrt{(Tr(J){)}^{2}-4|J|}). & \end{array}$$

In the absence of spatial variations, stability is essential for our system. To achieve this, it is sufficient for the steady-state solutions to be asymptotically stable, which requires that the real part of the eigenvalue λ of J of the linearized system is less than zero. Now, to ensure that Re(λ)<0 , the following conditions must be satisfied [36,39,58]:

(B 12)
$$\begin{array}{lrlr} & Tr(J)= & f_{u}+g_{v}<0, & \end{array}$$

(B 13)
$$\begin{array}{ll} |J|= & f_{u}g_{v}-f_{v}g_{u}>0. \end{array}$$

## Appendix C. Linear stability analysis of the full reaction-diffusion system

In §2.2, we identified the regions in which the uniform steady state $\left(u^{*},v^{*}\right)$ is stable in the absence of diffusion. Now, consider the full reaction-diffusion system and carry out linear stability analysis. We substitute the small perturbations given by

(C 1)
$$\begin{array}{lcr} & u(x,t)=u^{*}+\epsilon w_{1}(x,t),\;\text{and}\;v(x,t)=v^{*}+\epsilon w_{2}(x,t), & \end{array}$$

into the reaction-diffusion system (2.1), resulting in the linearized system:

$$\begin{array}{r} \frac{\partial }{\partial t}\left(\begin{array}{c} w_{1}\\ w_{2} \end{array}\right)={\left(\begin{array}{cc} f_{u} & f_{v}\\ g_{u} & g_{v} \end{array}\right)}_{\left(u^{*},v^{*}\right)}\left(\begin{array}{c} w_{1}\\ w_{2} \end{array}\right)+\left(\begin{array}{cc} 1 & 0\\ 0 & d \end{array}\right)\frac{\partial^{2}}{\partial x^{2}}\left(\begin{array}{c} w_{1}\\ w_{2} \end{array}\right), \end{array}$$

for the o(ϵ) terms only. The o(1) terms satisfy the equations for the homogeneous solution. $O(\epsilon^{2}$ ) and higher terms are ignored. The above equation can be written in matrix-vector form as:

(C 2)
$$\begin{array}{lrr} & w_{t}=\gamma Jw+D\nabla^{2}w, & \end{array}$$

where $J=\left(\begin{array}{cc} f_{u} & f_{v}\\ g_{u} & g_{v} \end{array}\right)$ , $D=\left(\begin{array}{cc} 1 & 0\\ 0 & d \end{array}\right)$ and $w=\left(\begin{array}{c} w_{1}\\ w_{2} \end{array}\right)$ .

We observe that equation (C 2) is a linear parabolic PDE whose solution, obtained through separation of variables, is given by:

(C 3)
$$\begin{array}{lrr} & w(x,t)=\sum_{k}c_{k}e^{\lambda t}\Phi_{k}(x). & \end{array}$$

For each wave number k , we find the vector of Fourier coefficients $c_{k}$ and $\Phi_{k}$ , the eigenfunction of the Laplacian, which satisfies

(C 4)
$$\begin{array}{l} \nabla^{2}\Phi_{k}+k^{2}\Phi_{k}=0,\;\text{for}\;x\in \Omega , \end{array}$$

subject to zero-flux boundary conditions

(C 5)
$$\begin{array}{l} (n.\nabla )\Phi_{k}=0,\;\text{for}\;x\in \partial \Omega , \end{array}$$

where n is the unit outward normal vector on the boundary ∂Ω. Substituting (C 3) and (C 4 ) into (C 2) yields for each k , the eigenvalue problem

(C 6)
$$\begin{array}{lrr} & (\lambda I-\gamma J+Dk^{2})c_{k}=0, & \end{array}$$

where I is the identity matrix. For non-trivial solutions for $c_{k}$ , we require that

$$\begin{array}{l} |\lambda I-\gamma J+Dk^{2}|=\left|\begin{array}{cc} \lambda -\gamma f_{u}+k^{2} & \gamma f_{v}\\ \gamma g_{u} & \lambda -\gamma g_{v}+dk^{2} \end{array}\right|=0, \end{array}$$

from which $\lambda =\lambda (k^{2})$ are the roots of the characteristic equation

(C 7)
$$\begin{array}{lrr} & \lambda^{2}+p(k^{2})\lambda +q(k^{2})=0, & \end{array}$$

where

(C8)p(k2)=k2(1+d)−γ(fu+gv),(C9)q(k2)=dk4−γ(dfu+gv)k2+γ2(fugv−fvgu).

DDI occurs when one of the roots of (C 7) have Re $\lambda (k^{2})>0$ for some $k^{2}>0.$ The Re $\lambda (k^{2})>0$ if either $p(k^{2})<0$ or $q(k^{2})<0$ for some $k^{2}>0$ . By condition (C 12), $p(k^{2})>0$ for all $k^{2}>0$ . Hence, we require that $q(k^{2})<0$ , for some $k^{2}>0$ , and this holds if

$$df_{u}+gv>0,$$

which gives us the first condition involving diffusion. Since, we are seeking real wave numbers, $k^{2}$ , we can find these wave numbers explicitly by solving the quadratic equation $q(k^{2})=0$ to obtain

$$k_{r,l}^{2}=\frac{\gamma }{2d}\left((df_{u}+g_{v})\pm \sqrt{\left(df_{u}+g_{v}{)}^{2}-4d(f_{u}g_{v}-f_{v}g_{u}\right)}\right).$$

Hence, $k_{r,l}^{2}$ are real if and only if

(C 10)
$$\begin{array}{lrr} & \frac{(df_{u}+g_{v}{)}^{2}}{4d}>f_{u}g_{v}-f_{v}g_{u}, & \end{array}$$

which yields the second condition involving diffusion.

In summary, the conditions for DDI [36,49,52] are given by:

(C 11)
$$\begin{array}{lrlr} & \text{without diffusion} & \left\{ \begin{array}{c} f_{u}+g_{v}<0,\\ f_{u}g_{v}-f_{v}g_{u}>0, \end{array}\right. & \end{array}$$

(C 12)
$$\begin{array}{lrlr} & \text{with diffusion} & \left\{ \begin{array}{c} df_{u}+g_{v}>0,\\ (df_{u}+g_{v}{)}^{2}-4d(f_{u}g_{v}-f_{v}g_{u})>0. \end{array}\right. & \end{array}$$

Inequalities (C 11) and (C 12) define the parameter space known as the Turing space. That is, a region in which a uniform steady state stable in the absence of diffusion is driven unstable in the presence of diffusion. By substituting (C 12) into $df_{u}+g_{v}>0$ , and assuming that u is an activator, that is, $f_{u}>0$ , then we obtain a constraint on d for DDI, that is

(C 13)
$$\begin{array}{lrr} & df_{u}-g_{v}>0\;⟹\;f_{u}(d-1)>0\;⟹\;d>1. & \end{array}$$

For Turing DDI in a 2-species component RDE in stationary domains, we require that the inhibitor diffuses a lot faster than the activator; that is, we require a long-range inhibition, short-range activation mechanism [27]. However, an n coupled RDE, this condition is not necessary [59,60].

For $q(k^{2})<0$ in (C 9) for some k>0 , the minimum value of $q(k^{2})$ , denoted as $q(k^{2}{)}_{min}$ must be negative. This is determined by setting the derivative $q^{\prime }(k^{2})$ to zero and solving for $k^{2}$ . Thus from,

$$\begin{array}{lrr} & q^{\prime }(k^{2})=2dk^{2}-\gamma (df_{u}+g_{v})=0, & \end{array}$$

then

(C 14)
$$\begin{array}{lcr} & k^{2}=k_{min}^{2}=\gamma \frac{\left[df_{u}-g_{v}\right]}{2d}. & \end{array}$$

This yields

$$\begin{array}{rl} q(k^{2}{)}_{min}= & \gamma [d(\frac{df_{u}+g_{v}}{2d}{)}^{2}-(df_{u}+g_{v})\frac{\left(df_{u}+g_{v}\right)}{2d}+|J|],\\ = & \gamma^{2}[|J|-\frac{(df_{u}+g_{v}{)}^{2}}{4d}]. \end{array}$$

For $q(k^{2})<0$ in (C 9), the necessary condition is

(C 15)
$$\begin{array}{r} |J|<\frac{(df_{u}+g_{v}{)}^{2}}{4d}. \end{array}$$

This is exactly, the last condition for Turing diffusion-driven instability (C 10).

Bifurcation occurs when $q(k^{2}{)}_{min}=0$ , hence

$$|J|=\frac{(df_{u}+g_{v}{)}^{2}}{4d}.$$

The equation provided above is a quadratic equation with respect to the variable d . When all parameter values are held constant, we can determine a critical diffusion coefficient, $d_{c}$ , which is the valid solution to the quadratic equation:

(C 16)
$$\begin{array}{lrr} & d_{c}^{2}f_{u}^{2}+2d_{c}(2f_{v}g_{u}-f_{u}g_{v})+g_{v}^{2}=0. & \end{array}$$

The solutions to (C 16) can be expressed as:

(C 17)
$$\begin{array}{lrr} & d_{c}=\frac{-(2f_{v}g_{u}-f_{u}g_{v})\pm \sqrt{(2f_{v}g_{u}-f_{u}g_{v}{)}^{2}-f_{u}^{2}g_{v}^{2}}}{f_{u}^{2}}. & \end{array}$$

In relation to the critical diffusion coefficient $d_{c},$ there is a corresponding wavenumber $k_{c}^{2}$ that satisfies equation (C 9). This is found by employing basic calculus to obtain

(C 18)
$$\begin{array}{r} k_{c}^{2}=\frac{d_{c}f_{u}+g_{v}}{2d_{c}}=\sqrt{\frac{|J|}{d_{c}}}=\sqrt{\frac{f_{u}g_{v}-f_{v}g_{u}}{d_{c}}}. \end{array}$$

The steady state $\left(u^{*},v^{*}\right)$ becomes linearly unstable when the diffusion ratio, d , exceeds a critical value represented by $d_{c}$ . This condition implies that $d_{c}$ serves as a bifurcation point, marking the onset of diffusion driven instability in the system. According to figure 21, it is evident that there is a specific range of values of $k^{2}$ such that $k_{l}^{2}<k^{2}<k_{r}^{2}$ where $q(k^{2})<0$ and $Re(\lambda (k^{2}))>0$ with

(C 19)
$$\begin{array}{lrr} & w(x,t)=\sum_{k}c_{k}e^{\lambda t}\Phi_{k}(x),t\to \infty . & \end{array}$$

The values $k_{l}^{2}$ and $k_{r}^{2}$ are the roots of $q(k^{2})=0$ and are explicitly provided in equations (C 20a) and (C 20b) respectively. Observing the unstable range of values of $k^{2}$ , it becomes evident that when the domain size, denoted by $L_{x}$ , is relatively small, no excitable wave modes fall within this range. Consequently, under such conditions, pattern formation cannot occur [36]. As a consequence, there exist values of $q(k^{2})$ where $q(k^{2})<0$ and $Re(\lambda (k^{2}))>0$ for $k_{l}^{2}<k_{c}^{2}<k_{r}^{2}$ , where

(C 20a)
$$\begin{array}{lrlr} & k_{l}^{2}= & \frac{\gamma }{2d}[(df_{u}+g_{v})-\sqrt{(df_{u}+g_{v}{)}^{2}-4d|J|}], & \end{array}$$

(C 20b)
[Disp-formula A1-E20]
$$\begin{array}{lrlr} & k_{r}^{2}= & \frac{\gamma }{2d}[(df_{u}+g_{v})+\sqrt{(df_{u}+g_{v}{)}^{2}-4d|J|}], & \end{array}$$

correspond to the roots of the equation $q(k^{2})=0$ . We use this spectrum to identify specific modes that can be isolated. The unstable modes are associated with the eigenfunctions of the Laplacian on the domain Ω , taking into account the boundary conditions (2.1).

## Appendix D. Isolation of wave numbers in one-dimension

When finding the solution to equation (C 2), we take guidance from Murray’s work [36]. For each value of k , we seek solutions that satisfy the equation

(D 1)
$$\begin{array}{r} \nabla^{2}\Phi_{k}+k^{2}\Phi_{k}=0,\;x\in \Omega , \end{array}$$

subject to homogeneous zero-flux boundary conditions

(D 2)
$$\begin{array}{r} \left(n\cdot \nabla \right)\Phi_{k}=0,\;x\in \partial \Omega . \end{array}$$

In this section, our focus revolves around exploring solutions to equations (D 1) and (D 2) within a one-dimensional context. The solutions we derive will serve as a benchmark for assessing the accuracy and reliability of the numerical techniques we use. Equation (D 1) with boundary conditions (D 2) on a one-dimensional domain $\left[0,L_{x}\right]$ has a solution given by

(D 3)
$$\begin{array}{r} \Phi_{n}(x)=\cos\left(\frac{n\pi x}{L_{x}}\right),\;\text{ for }\;n=1,2,\cdots . \end{array}$$

In this context, the permissible wave numbers take the form $k_{n}=\frac{n\pi }{L_{x}}$ . The general solution of (C 2) is therefore given by

(D 4)
$$\begin{array}{r} w(t,x)=\sum_{k}c_{k}e^{\lambda t}\Phi_{k}(x)=0. \end{array}$$

We consider a domain $L_{x}$ of unit length in one dimension in equation (D 3). The general form of eigenvalues has been shown to be of the form $k_{n}^{2}=n^{2}\pi^{2}$ [52]. Turing instability occurs when conditions (C 11) and (C 12) are satisfied. In addition, we have the following relationship:

(D 5)
$$\begin{array}{lrr} & \gamma B_{1}=k_{l}^{2}<k_{n}^{2}<k_{r}^{2}=\gamma B_{2}, & \end{array}$$

where $B_{1}$ and $B_{2}$ are solutions to equation (C 9). It is crucial to stress that equation (D 5) serves as a necessary condition, but is insufficient alone to uniquely determine a specific wave number. In addition to (D 5), we require the condition expressed in equation (D 6):

(D 6)
$$\begin{array}{lrr} & k_{n-1}^{2}<k_{l}^{2}<k_{n}^{2}<k_{r}^{2}<k_{n+1}^{2}. & \end{array}$$

We observe that as the domain size $L_{x}$ increases, it eventually reaches a critical bifurcation value, at which point an increasing number of distinct wave numbers start to appear, and subsequently, patterns begin to form. It must be noted that increasing γ is equivalent to increasing the domain size. Hence, re-arranging equation (C 14), we can compute the minimum value of γ using the relation

(D 7)
$$\begin{array}{lrr} & \gamma =\frac{2d_{c}k^{2}}{d_{c}f_{u}+g_{v}}. & \end{array}$$

Here, we take $d_{c}=5.84$ , $f_{u}=0.0798$ , $g_{v}=-0.1547$ , as computed before, and the first mode $k^{2}=\pi^{2}$ to compute the critical $\gamma_{c}$ beyond which we excite this first mode. That is:

$$\begin{array}{r} \gamma =\frac{2\times 5.84\times k^{2}}{5.84\times 0.0798-0.1547}=370.2. \end{array}$$

Fixing d=10 and plotting the dispersion relations for γ=200 , γ=1500 and γ=5000 , we observe that the number of excited modes increases as shown in figure 22. A summary of the excited wave numbers for d=10 and a selected range of values of γ is provided in table 7.

**Table 7.** The range of values for γ associated with excited wave numbers isolated for d=10 .

γ	excitable wave numbers isolated	mode count
0−172	—	0
173−691	$k_{1}=\pi$	1
692–1356	$k_{1}=\pi$ , $k_{2}=2\pi$	2
1357– 1556	$k_{2}=2\pi$	1
1557−2767	$k_{2}=2\pi$ , $k_{3}=3\pi$	2
2768−4324	$k_{2}=2\pi$ , $k_{3}=3\pi$ , $k_{4}=4\pi$ , $k_{5}=5\pi$	3
4324−5423	$k_{3}=3\pi$ , $k_{4}=4\pi$ , $k_{5}=5\pi$	4
5424−6227	$k_{3}=3\pi$ , $k_{4}=4\pi$ , $k_{5}=5\pi$	3
6227−8476	$k_{3}=3\pi$ $k_{4}=4\pi$ , $k_{5}=5\pi$ , $k_{6}=6\pi$	4
8477−10000	$k_{3}=3\pi$ , $k_{4}=4\pi$ , $k_{5}=5\pi$ , $k_{6}=6\pi$ , $;k_{7}=7\pi$	5
